# Designing polyphosphazene derivatives for gene delivery in glioblastoma treatment

**DOI:** 10.1016/j.mtbio.2025.102010

**Published:** 2025-06-25

**Authors:** Carla Garcia-Mazas, Elia Bozzato, Jhonathan Angel Araujo Fernandez, Federico Quattrini, Veronique Preat, Laura Sanchez, Noemi Csaba, Marcos Garcia-Fuentes

**Affiliations:** a Department of Pharmacology, Pharmacy and Pharmaceutical Technology, CiMUS Research Center and Health Research Institute of Santiago de Compostela (IDIS), University of Santiago de Compostela, Santiago de Compostela, 15706, Spain; b UCLouvain, Advanced Drug Delivery and Biomaterials Research Group, Louvain Drug Research Institute (LDRI), Avenue Mounier 73/B1.73.12, 1200, Brussels, Belgium; c Zebrafish Laboratory, Department of Translational Medicine, FCM, UNICAMP, Campinas, Brazil; d Department of Zoology, Genetics & Physical Anthropology, University of Santiago de Compostela, 27002, Lugo, Spain

**Keywords:** Polyphosphazenes, Glioblastoma, Gene delivery, Advanced therapies, Smart polymers, BMP

## Abstract

Gene therapy presents promising opportunities to target critical pathways in complex cancers like glioblastoma multiforme, though it necessitates the use of efficient delivery vectors. Polyphosphazenes (PPZs) are highly flexible materials that lead to biodegradable, high-performance materials in various applications, including gene delivery.

In this work, we synthesized various PPZ derivatives, incorporating primary amines, secondary amines, hydrophilic, and hydrophobic groups, and evaluated their gene transfection capabilities in combination with an anionic polyphosphazene (6MHA-PPZ) that acts as a charge quencher and transfection enhancer. Combining 6MHA-PPZ with a hydrophobic polymer demonstrated the highest gene delivery efficiency and safety, significantly surpassing previous benchmarks.

Using these optimized nanoparticles, we delivered a BMP4-expressing plasmid (pBMP4) in glioblastoma models. The pBMP4 nanoparticles, when combined with the chemotherapeutic agent temozolomide (Tz), resulted in significant reductions in tumor volume, improved survival rates in preclinical models, and normalized the expression of drug resistance markers, providing a synergistic antitumoral effect with Tz.

This study highlights the potential of PPZ-based nanoparticles for gene delivery and suggests that the combination of pBMP4-NPs and Tz could offer a promising therapeutic strategy for treating glioblastoma.

## Graphical abstract

**Figure:**
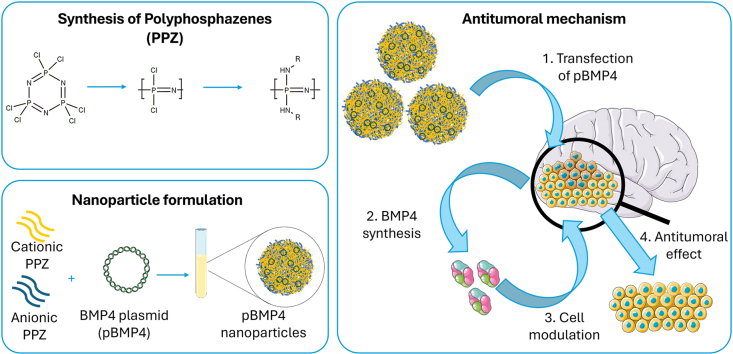

## 1 Introduction

Gene therapy has emerged as a promising strategy to modify the tumor microenvironment and target key cellular functions in resistant cancer cells. Pathways such as TGFβ, Hedgehog, Wnt, and NF-kB are critical targets for cancer suppression [1]. For example, BMPs, part of the TGFβ superfamily, are known to suppress glioblastoma-initiating cells. These pathways can be modulated by conventional drugs or proteins, but gene therapy often provides more effective means of manipulation.

The success of gene therapies largely depends on the efficient delivery of polynucleotide sequences into the target cells. While lipid nanoparticles have become the standard vehicle for these therapies, polymeric nanoparticles offer advantages in terms of stability and structural flexibility [2]. In addition, as new applications for gene therapy are explored, there is a growing need to investigate alternative delivery platforms [3].

Polyphosphazenes (PPZs) are biodegradable, highly flexible polymers that are being increasingly applied in various biomedical fields [4]. PPZ derivatives have been synthesized to function as delivery systems for gene therapy with successful results. These PPZ are most frequently modified with tertiary amine groups, such as 2-dimethylaminoethanol [5]. To improve their characteristics, polymers have also been further modified with groups that favor endosomal escape (e.g., imidazole) and improve targeting and biocompatibility (e.g., galactose or polyethylene glycol) [6,7]. Recently, we published a click-chemistry approach to generate several PPZ derivatives for gene delivery, demonstrating that PPZ derivatives with primary amines had better gene transfer characteristics than those with tertiary amines [8]. Additionally, we designed and characterized a specific anionic PPZ with 6-mercaptohexanoic acid (6MHA) side groups. Nanoparticles containing 6MHA-PPZ showed lower toxicity and enhanced gene delivery efficiency due to a pH-dependent membrane destabilisation mechanism [8]. These initial data indicate potential pathways for designing PPZ-based gene delivery systems. However, further studies are necessary to achieve a rational design of these.

Poor clinical outcomes in glioblastoma treatment are thought to stem from a subset of chemotherapy-resistant cells known as glioblastoma initiating cells, which are capable of initiating tumor regrowth. Both academic researchers and the pharmaceutical industry have been actively seeking drugs to specifically target these tumor-initiating cells. Bone Morphogenetic Proteins (BMPs), which are morphogens in the TGFβ superfamily, have shown promise in suppressing glioblastoma initiating cells, likely by reprogramming them into terminally differentiated phenotypes [1,9].

In the present study, we introduce and evaluate three novel polyphosphazene derivatives, synthesized and investigated here for the first time as gene delivery vectors. These heteropolymers, incorporating hydrophobic, secondary amine, and hydrophilic functionalities respectively, were thoroughly characterized and tested for their physicochemical properties, biocompatibility, and transfection efficiency. Among them, the polymer with both amine and hydrophobic groups demonstrated outstanding performance, surpassing both commercial benchmarks and previously reported prototypes from our group. From a therapeutic perspective, this study also pioneers the use of polyphosphazene-based nanocarriers for the delivery of BMP-encoding plasmids in glioblastoma models, offering a novel and promising strategy for targeting this aggressive cancer.

## 2 Materials and methods

### 2.1 Materials

Aluminum chloride (99.99 %), Anhydrous tetrahydrofuran (THF), Cysteamine (Cys), Chloroform-d (99.96 atom % D containing 0.03 % TMS), Deuterium oxide (99.9 atom % D, containing 0.05 wt % 3-(trimethylsilyl)propionic-2,2,3,3-d4 acid), Heparin sodium salt (from porcine intestinal mucosa), HEPES (≥99.5 %), Hexachlorocyclotriphosphazene ((NPCl_2_)_3_) (99 %), Potassium chloride (BioXtra ≥99 %), Triethylamine (TEA), Tris-Acetate-EDTA buffer (10x), 1-mercapto-2-propanol (MP), 2-butylamino)ethanethiol (BET), 2-(dimethylamine)ethanethiol hydrochloride (DMAES), 2,2,2-trifluoroethanol (TFE), 2,2-dimethoxy-2-phenylacetophenone (DMPA), 2-methyl-1-propanethiol (MPT), 6-mercaptohexanoic acid (6MHA), Temozolomide and Xylazine were all purchased from Sigma-Aldrich. DNAse/RNAse free water, Dulbecco's Modified Eagle Medium (DMEM), OptiMEM, Eagle's minimal essential medium (EMEM), Fetal Bovine Serum (FBS), Penicillin-Streptomycin for culture medium, Dialysis membrane (molar mass cut-off 7 kDa), Lipofectamine 2000 Transfection reagent, SYBR® Gold nucleic acid stain, TRI-Reagent, RevertAid First Strand cDNA Synthesis Kit and lyophilized primers were purchased from ThermoFisher (USA). Ethanol, Crystal violet and acetic acid (glacial) were provided by Merk (Germany). Bone Morphogenic Protein 4 (BMP-4) was purchased from Peprotech (UK). Bio-Rad Protein Assay was provided by BioRad (USA). Luciferase Reporter Gene Assay was bought from Roche (Germany). MTS Cell Proliferation Assay Kit was purchased to BioVision (USA), Alamar Blue were bought to Promega (Spain). NZYSpeedy qPCR Green Master Mix (2x) was purchased from NZYTech (Portugal). Ketamine for the anesthesia was provided by Pfizer (USA). BMP4 plasmid was bought from Sino Biological Inc. (China). The pEGFPLuc plasmid was donated by Prof. Anxo Vidal's laboratory (CiMUS, Universidad de Santiago de Compostela) and amplified in-house (S1). All the products were used as received.

### 2.2 Synthesis of the precursor poly(allylamino-phosphazene) (AAPPZ)

The precursor synthesis was carried out following the protocol previously developed in our lab [8]. Briefly, in a previously dried flask 14.4 mmol of hexachlorocyclophosphazene ((NPCl_2_)_3_) was mixed with 7.5 % aluminum chloride (w/w) (catalyst) in an inert atmosphere of nitrogen and heated at 240–250 °C for 3 h. After the polymerization, the product was cooled to 120 °C and solubilized in diglyme to minimize crosslinking and avoid the solidification of the crude product. Then, it was centrifuged to remove the aluminum chloride (−10 °C, 7000 rcf, 5 min) and the supernatant was transferred to a flask with anhydrous THF, TEA and allylamine (both 3 eq to chlorine). This reaction was maintained in an ice bath for one day, and then another day at room temperature. The resulting product was filtered to remove TEA hydrochloride and subsequently precipitated with water, centrifuged (4 °C, 7000G, 10 min) and the precipitate was collected and dried under vacuum overnight. AAPPZ was characterized by ^31^P, ^1^H NMR and DOSY.

### 2.3 Precursor radical modification by thiol-ene click chemistry

**Fig. 1:**
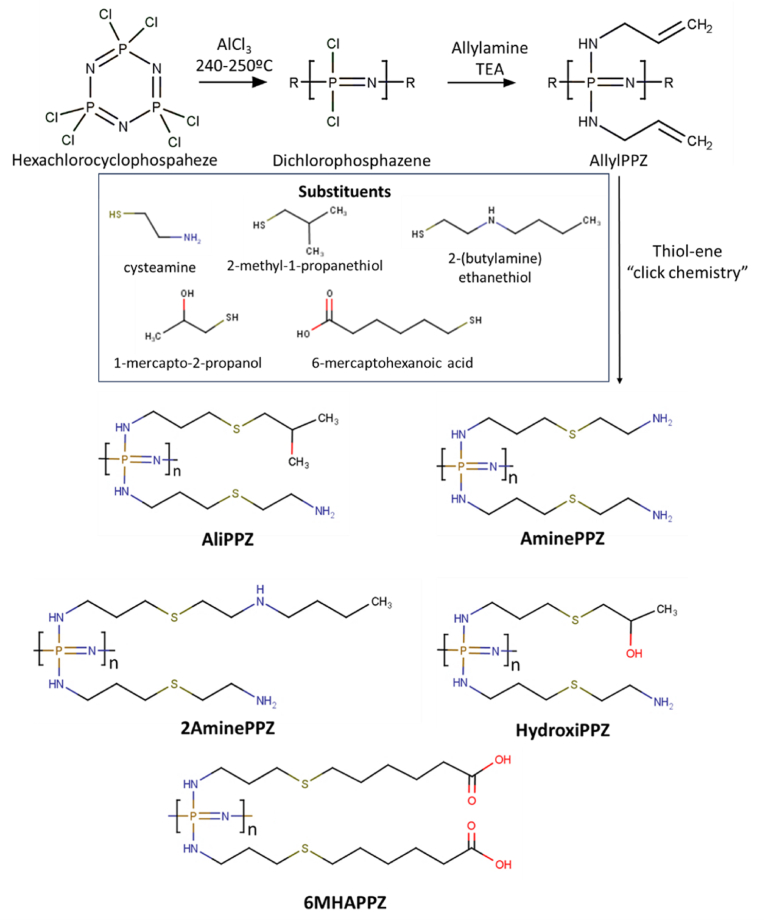
Scheme of polyphosphazene modification by thiol-ene click chemistry.

The side chains of AAPPZ were modified by thiol-ene click chemistry to introduce different radicals and the thiol group of the new compounds reacted with the allyl group of the AAPPZ obtaining five different polyphosphazenes (Fig. 1). Briefly, poly(allylamino-phosphazene) (AAPPZ) was dissolved in trifluoroethanol (TFE) and mixed with the desired substituent (3 eq to allyl group): cysteamine, 1-mercapto-2-propanol (MP), 2-(butylamine) ethanethiol (BET), 2-methyl-1-propanethiol (MPT), and 6-mercaptohexanoic acid (6MHA). The mixture was bubbled with nitrogen, and the catalyst 2,2-dimethoxy-2-phenylacetophenone (DMAES) (0.05 eq to allyl group) was added to the reaction mixture. The reaction proceeded under magnetic stirring and UV irradiation (λ = 365 nm) for 3 h. The resulting product was purified by dialysis (membrane molar mass cut-off 7 kDa) against HCl 2 mM for 24 h and 48 h against water. The dialysate was freeze-dried and then analyzed by ^31^P and ^1^H NMR, COSY and HSQC. AminePPZ was used as a control for all the experiments.

### 2.4 Polymer characterization

#### 2.4.1 Nuclear magnetic Resonance spectroscopy (NMR)

^1^H and ^31^P NMR spectra were obtained by Bruker 400 and DRX-500 spectrometers operated by RIAIDT (University of Santiago de Compostela). For the bidimensional NMR (COSY and HSQC) were recorded by Varian Mercury 300 spectrometer. Solvents used for polymer dissolutions were CDCl_3_ and D_2_O and all chemical shifts are reported in parts per million (ppm) relative to tetramethylsilane (TMS) or known solvent peak positions.

#### 2.4.2 Determination of polymer molecular weight

To determine the molar masses, polymers were dissolved in NaCl 10 mM at 5 mg/mL and measured by Asymmetric Flow Field-Flow Fractionation (AF4), using an AF2000 MultiFlow FFF coupled to a Multi-Angle Light Scattering (MALS) (Postnova, Germany) detector. MALS was normalized with a BSA standard monomer (66 kDa) and quality control was performed daily with Pullulan standard (48.8 kDa).

### 2.5 Nanoparticle formation

The nanoparticles were prepared by ionic complexation, using the model plasmid (pEGFPLuc) or therapeutic plasmid (pBMP4). The polymers were dissolved in HEPES 10 mM (pH 5.5) and the pDNA in pure water. Nanoparticles were formed upon electrostatic interaction of pDNA or pDNA/anionic polymer mixtures, with the cationic polymer; the preparation was performed under magnetic stirring (500 rpm, 5min). Different component ratios were investigated. For nanoparticles containing cationic polymer and pDNA, the ratios are based on the number of primary/secondary amines of the polymer branches (N) and the phosphates of the pDNA (P). In this case, composition is defined as N:P ratio. In the case of nanoparticles that contain the anionic polymer 6MHA-PPZ, the amount of this material is quantified by the number of terminal carboxylic groups (C), and the composition of the nanoparticles is defined by the N:C:P ratio.

### 2.6 Nanoparticle characterization

#### 2.6.1 Size, zeta potential and concentration of nanoparticles

The nanoparticles were characterized in terms of size, by Dynamic Light Scattering (DLS), and in terms of Zeta Potential, by laser doppler anemometry (Nanosizer ZS, Malvern, UK). Each analysis was performed in triplicate at 25 °C, with a backscatter angle of 173°. Nanoparticle hydrodynamic diameter, distribution and concentration were also determined through Nanoparticle Tracking Analysis (NTA) using a Nanosight NS300 system (Malvern Instruments, Worcestershire, UK) equipped with a laser operating at λ = 488 nm, after diluting the samples 1:400 in HEPES 10 mM. Zeta potential measurements were performed upon dilution 1:10 in 1 mM KCl.

#### 2.6.2 Morphological analysis of the nanoparticles

Morphological analysis of nanoparticles was done by Field Emission Scanning Electron Microscopy (FESEM) with Energy Dispersive X-ray spectroscopy (Zeiss Gemini Ultra Plus, Germany), using scanning transmission electron microscopy (STEM) and immersion lens (InLens) detectors for sample observation. For sample preparation, 10 μL of the nanoparticles were placed on a copper grid with carbon films and allowed to dry for 5 min; sample excess was removed by blotting. The sample was stained by adding the same volume of phosphotungstic acid (2 % w/w in water). Afterwards the staining was washed by blotting and washed twice with water. Once dried, the sample were observed through STEM and immersion lens (InLens) detectors.

#### 2.6.3 Binding efficiency of nanoparticles

The nucleic acid binding efficiency of the nanoparticles was determined by gel retardation assay. The samples were loaded in an agarose gel (1 % w/v in Tris-EDTA 1x buffer). Each well contained 0.33 μg of pDNA, and free pDNA was used as control. For sample loading and visualization, all the samples contained 1 x SYBR® Gold nucleic acid stain and loading buffer (30 % glycerol and 0.25 % bromophenol blue). The dissociation assay was performed by incubating samples with an excess of an anionic competitor (20:1 w/w heparin: pDNA) for 1 h at 37 °C.

### 2.7 Cell culture

*In vitro* assays were performed in U87MG and U251 glioblastoma cell models. Cells were grown in Dulbecco's Modified Eagle Medium (DMEM) or Eagle's minimal essential medium (EMEM), in the case of nanoparticles containing the therapeutic plasmid, medium was supplemented with 10 % (v/v) heat inactivated Fetal Bovine Serum (FBS) and 1 % (v/v) Penicillin/Streptomycin (P/S) and incubated at 37 °C (95 % relative humidity and 5 % CO_2_) up to 85 % confluence, when they were subcultured by trypsinization, dilution and plating as described below.

### 2.8 *In vitro* toxicity

For 2D toxicity assays, 8000 cells/well were seeded on a 96-multiwell plate and incubated 24 h before the treatment to allow cell attachment. Then, the nanoparticles were incubated in supplemented medium for 4 h at different pDNA concentrations (0.1–2 μg pDNA/cm^2^). After nanoparticle incubation, the medium was removed and replaced with fresh one, and cells were allowed to recuperate for 48 h. The cytotoxicity evaluation was performed adding 10 μl of MTS per well and the absorbance was measured after 3 h of incubation in a plate reader at 495 nm. LC50 was calculated using a logistic regression (GraphPad Prism).

For the 3D toxicity assay, the nanoparticles were incubated with neurospheres. To form neurospheres, 300 U87MG cells/well were seeded on a ULA 96-multiwell plate (Ultra Low Attachment) by centrifugation (20 min, 200 rcf). After 3 days, the nanoparticles were incubated for 12 h at 2 μg pDNA/mL nanoparticle concentration. After 12 h the nanoparticle suspensions were replaced with fresh medium and the neurospheres were incubated for 72 h additional hours. Cell toxicity was quantified based on two parameters: (1) evolution on neurosphere size and shape at 0, 24, 48 and 72 h and (2) a colorimetric readout at 72 h in a resazurin reduction assay (CellTiter-Blue®, Promega, USA) that measures cell metabolism. For the CellTiter-Blue assay, 40 μl of the reagent was added per well and incubated for 4 h with the cells. The fluorescence was evaluated on a plate reader at 539 nm of excitation wavelength and 620 nm of emission.

In all of the cytotoxicity tests, the negative control (no toxicity) was HEPES 10 mM and the positive control (100 % toxicity) was Triton 0.1 %.

### 2.9 Fish Embryo Acute Toxicity test (FET)

The wild type zebrafish were housed in a water recirculation system under controlled physicochemical conditions of temperature, pH and conductivity of 26 ± 2 °C, 7–7.5 and 400–600 μS/cm respectively [10]. The experimental design has been carried out based on the OECD protocol (Organization for Economic Cooperation and Development) for the study of toxicity in fish embryos known as Fish Embryo Acute Toxicity (FET) test [11]. The fertilized eggs were collected as soon as possible after natural spawning (stages of 16–32 cells) [12]. Twenty fertilized eggs per group, five concentrations tested for nanoparticles, were placed in 96-well plate. Embryos were incubated at 26 ± 2 °C for 96 h and were observed on an inverted microscope every 24 h, looking for toxicity signs. The observations performed to determine the toxicity include the detection of coagulation of embryos, lack of somite formation, non-detachment of tail, lack of heartbeat (after 48 h) or oedema in the embryo (Fig. S2).

To consider the experiments valid, in the negative control there must be a mortality rate ≤10 % with a hatching rate> 80 % at the end of the test (96 h postfertilization; hpf).

All experiments and protocols were approved by the animal care and use committee of the University of Santiago de Compostela and the standard protocols of Spain (CEEA-LU-003 and Directive 2012-63-EU). The toxicity evaluation data were analyzed by probit analysis using ToxRat software (ToxRat Solutions. 2003. ToxRat. Software for statistical analysis of bioassays. Alsdorf, Germany).

### 2.10 *In vitro* transfection

The pGFP-luc plasmid was used for transfection experiments (see structure in Fig. S1). This plasmid co-expresses eGFP (enhanced green fluorescent protein) that stains the cells (qualitative result), and luciferase that in the presence of its substrate luciferin provides quantifiable results. Cell transfection was validated by fluorescence microscopy, observing eGFP staining.

In this case 56,000 U87MG cells/well were seeded on a 24-multiwell plate in DMEM medium supplemented with 10 % FBS and 1 % P/S. After 24 h, nanoparticles were added at 0.5 μg de pDNA/cm^2^ in OptiMEM (Gibco, USA) medium and incubated for 4 h. Afterwards, the nanoparticles were washed, and the medium was replaced by supplemented DMEM. The cells were allowed to grow for 48 h.

To quantify transfection, we measured luciferase expression by a Luciferase Reporter Gene Assay (Roche, Germany). Briefly, the cells were washed twice with PBS and 100 μL of lysis buffer was added. After 5 min the lysate was centrifuged, and 50 μL of the supernatant was collected and placed on a white plate and using an automatic injector. Then, 25 μL of luciferin from the commercial kit was added to the sample, before measurement in a luminometer (Mithras LB 940, Berthold).

The results were corrected for protein content, quantified by a Bio-Rad Protein Assay. For this assay, 40 μL of the reagent was added to the sample and the absorbance was measured spectrophotometrically at 595 nm. The luminescence was expressed as relative luminescence units (RLU) per microgram of protein. Lipofectamine 2000, used following the manufacturer's instructions, was used as positive control.

### 2.11 *In vitro* antitumoral effect

#### 2.11.1 Clonogenicity assay

Two lines of human glioblastoma cells, U87MG and U251, were used in this experiment. Both cell lines were cultivated in supplemented EMEM medium. For antitumoral experiments, a plasmid encoding for the Bone Morphogenetic Protein-4 was loaded into the AliPPZ 6:4:1 nanoparticles (pBMP4-NPs). The structure of the plasmid is depicted in Fig. S1.

For the clonogenicity assay, 10^5^ cells/well were seeded in a 12-multiwell plate. After 24 h the treatment was added, the cells were treated with seven different treatments: Medium, BMP4 protein, Blank-NPs, pBMP4-NPs, temozolomide (Tz), BMP4-protein + Tz combination, and pBMP-NPs + Tz combination. The concentration was 23.2 μg of nanoparticles/mL, 30 ng BMP4-protein/mL and 2.4 μg temozolomide/mL. Two days later, 500 cells/well were re-seeded on a 6-multiwell plate and allowed to grow for 12 additional days. The cells were stained with a preparation of 50 % ethanol, 5 % acetic acid and 0.5 % crystal violet, washed twice with water and the number of colonies was counted per each well.

#### 2.11.2 Evaluation of the treatment combination effect

The effect of the co-administration of the temozolomide and the BMP4 protein/pBMP4-NPs was evaluated by calculating the coefficient of drug interaction (CDI) using the following formula: CDI = AB/(A × B). AB is the number of colonies formed (fraction of the control group) from the combined effects of both treatments. A or B is the number of colonies formed (fraction of the control group) of each treatment separately. A CDI <1 indicates that the combination of treatments has a synergistic effect, while if CDI = 1 it is additive and if CDI >1, is antagonistic [13].

### 2.12 *In vivo* antitumor efficacy

#### 2.12.1 *In vivo* glioblastoma xenograft mouse model and treatment administration

The experiment was approved by the ethical animal care committee of the Université Catholique de Louvain (2019/UCL/MD004) and was performed according to the Belgian National Guidelines in accordance with European Directive. Animals had free access to water and food all the time.

Briefly, eight-week-old NMRI female nude mice (Janvier, France) were anesthetized intraperitoneally with 150 μL of a solution of 10 mg/ml ketamine and 1 mg/ml xylazine, and two million of fresh U87MG cells were administered subcutaneously in the flank. The tumor was allowed to grow for ten days before the administration of the treatment.

**Fig. 2:**
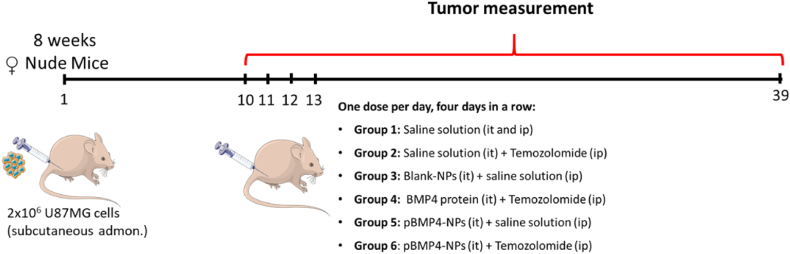
Scheme of the in vivo assay in glioblastoma xenograft model in 8-week-old female nude mice. The tumor was generated after the administration of 2x10^6^ cells U87MG/mouse. The treatment was administered after 10 days, when the tumors had an average volume of 35 mm^3^. Mice were divided into six groups and treated with four doses administered four days in a row. The evolution of the tumors and animal weight was studied until mice were sacrificed. NPs: nanoparticles; BMP4: Bone morphogenic protein 4; Tz: temozolomide; it: intratumoral; ip: intraperitoneal.

When the tumor size was around 35 mm^3^, mice were anesthetized, randomized in six groups and the treatment was administered intratumorally in four doses in four consecutive days. Experimental groups were: (Group 1) Control (n = 7); (Group 2) Temozolomide (Tz) (n = 6); (Group 3) Blank nanoparticles (n = 6); (Group 4) BMP4 + Tz (n = 6); (Group 5) pBMP4 nanoparticles (n = 7) and (Group 6) pBMP4 nanoparticles + Tz (n = 7). As control, we used saline solution (Fig. 2). For temozolomide, used as the standard chemotherapy used in the glioblastoma, the dose administered was 5 μg of temozolomide/g of animal, administered intraperitoneally as free drug. For nanoparticles and BMP4 protein the doses were 1.54 μg of BMP4-pDNA nanoparticles/g and 2 ng/g, respectively.

#### 2.12.2 Tumor growth and survival rate

After the administration of the different treatments, tumor size and body weight were measured every 2 days. Tumor size was measured by callipers and volume was calculated according to the following formula V = L x W x H, wherein L is the length, W is the width, and H is the height of the tumor. Relative tumor volume was calculated as V/Vo (Vo is the tumor volume before the first administration). Mice were sacrificed either when the tumor volume was higher than 1500 mm^3^, on the appearance of necrosis or ulcers, with >20 % weight loss or in presence of distress signs. The tumors were collected at this endpoint.

#### 2.12.3 Tumor RNA extraction and real time-PCR

For RNA extraction, tumors were homogenized in 1 ml of TRI-Reagent (ThermoFisher) by GentleMACS Dissociator (Miltenyi Biotec, Germany). The homogenized tumor was centrifuged to remove the fatty layer and tissue debris. The homogenizate was mixed with 200 μl chloroform/mL TRI-Reagent by repeated turning and left to stand for 15 min at room temperature. Then, samples were centrifuged, and the aqueous phase was transferred to an Eppendorf containing 500 μl of isopropanol/mL of TRI-Reagent, mixed by repeated turning, and cooled to −20 °C for 15 min. The samples were centrifuged, and the supernatant was decanted, the pellet was washed twice with ethanol and allowed to dry at room temperature and resuspended in DNAse/RNAse-free water. The concentration was determined by NanoDrop 2000 (ThermoFisher). RNA samples were reverse transcribed using the kit RevertAid First Strand cDNA Synthesis Kit (ThermoFisher). Real time-PCR (RT-PCR) was made using the kit NZYSpeedy qPCR Green Master Mix (2x) (NZYTech, Portugal). Both PCR was carried out using the Mastercycler Nexus (Eppendorf, Germany). GAPDH was used as a housekeeping gene to normalize gene expression. Primer sequences for the genes of interest (Sox2, Nestin, Oct4, Nanog, BMP4, MDR1 (P-gp), MRP1, and ABCG2) are shown in Table S3.

### 2.13 Statistical analysis

Data were represented as mean ± standard deviation (SD). Statistical differences were calculated using the Student's t-test, one-way and two-way ANOVA in combination with Tukey's or Sidak's multiple comparisons test. The significance was set to p < 0.05. All the experiments were repeated three times unless stated otherwise.

## 3 Results

### 3.1 Polymer synthesis and characterization

#### 3.1.1 Characterization of the polymer composition

New gene delivery systems were designed based on homopolyphosphazenes with primary amines (PPZ- NH_2_), and heteropolyphosphazenes with primary amines and other grafting groups with potential interest for gene delivery. As a first step, the materials were synthesized, isolated, and characterized by NMR.

Polymer syntheses were based on a general scheme previously reported by us [8]. In the first reaction, the precursor poly(dichlorophophazene) was synthesized by a ring opening reaction after heating hexa(chlorocyclophosphazene) ((NPCl_2_)_3_) at high temperature. This method developed by Sohn et al. [14] avoids the presence of solvents used in other traditional methods [15,16]. High temperatures and the presence of a catalyst (AlCl_3_) were used to reduce polymerization time, and to control polymer molecular weight [14,17,18]. In the second reaction, the chlorine side-groups of poly(dichlorophosphazene) were substituted with allylamine, following Allcock's classical reaction [16,19]. The resulting polymer, allylamine substituted polyphosphazene (AAPPZ), can be modified by thiol-ene click chemistry. AAPPZ is used here as the central secondary precursor for all the polymers produced (Fig. 1).

The ^31^P NMR spectra proved the successful conversion of the monomer into AAPPZ (Fig. S4a). The chemical shift for the one peak present on AAPPZ spectrum was 3.64 ppm, similar to that reported in the literature, and different to the values typical for poly(dichlorophosphazene) (−20 ppm) and hexa(chlorocyclophosphazene) (20 ppm) [20,21]. The same values were also previously reported in our previous work, where AAPPZ was also characterized [8]. The organic side groups of AAPPZ were also analyzed by ^1^H NMR (Fig. S4). The most characteristic peaks of AAPPZ were the vinyl group protons at 4.8 and 6 ppm (Fig. S4, groups “c” and “d”). Analysis by DOSY-NMR confirmed that all groups in the AAPPZ spectra are part of the same molecule (Fig. S4) and that this molecule has high molecular weight.

**Fig. 3:**
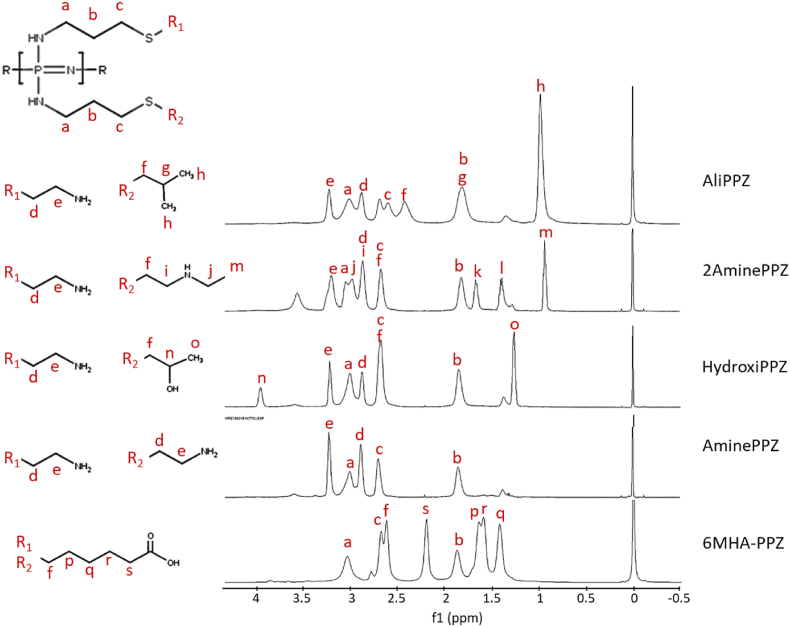
NMR-^1^H spectra of the synthetic polymers.

The last reaction was the substitution of AAPPZ with the different groups by thiol-ene addition (Fig. 1). The substituted polymers were also isolated by dialysis and characterized by NMR. ^31^P NMR of the modified polymers showed single peaks at similar chemical shifts than for AAPPZ (4.4–5 ppm) (Fig. S4b). The ^1^H NMR provided clear indications of side group substitution (Fig. 3). First, we could observe that the signal from the vinyl protons (4.8–6 ppm) disappears for all the cases, which suggests the complete addition of the thiol grafting groups. Conversely, other peaks indicative on the new substituted organic groups were observed.

Homopolyphosphazenes, AminePPZ and 6MHA-PPZ, had previously been synthetized and characterized, and the ^1^H NMR peaks obtained were consistent with this previous work [8] (Fig. 3, Fig. S4b and Fig. S4d). AliPPZ (AliphaticPPZ), 2AminePPZ and HydroxiPPZ are heteropolyphosphazenes containing the cysteamine group and a second grafting group, and they are reported here for the first time. The chemical composition of these new polymers was verified by analyzing ^1^H NMR (Figs. 3), ^1^H–^1^H COSY and ^1^H-^13^C HSQC 2D spectra (Fig. S4d). The most characteristic peak of AliPPZ is an intense signal at 1.0 ppm, attributed to the terminal methyl groups (peak “h”). A similar peak, but less intense, was observed for 2AminePPZ, but not for the other polymers (peak “m”). In this case, the key peaks are those proximal to the secondary amine (Fig. 3; peaks “i” and “j”). Unfortunately, these overlap with other peaks of the polymer, preventing adequate resolution. Therefore, bidimensional analysis by HMQC was applied for a more comprehensive identification (Fig. S4d). Regarding HydroxiPPZ, there are two characteristic peaks: one at 3.9 ppm, which can be attributed to the proton on the carbon of the hydroxyl group (Fig. 3; peak “n”), and another at 1.2 ppm that can be atributted to the terminal methyl group (Fig. 3; peak “o”).

Integration of the characteristic peaks of these heteropolyphosphazenes allowed us to quantify the proportion of the grafting radicals (Fig. 3; R_1_ and R_2_) for each heteropolymer. For AliPPZ we found a substitution close to 50 % for each substituent. In 2AminePPZ the substitution is 68 % of the cysteamine radical and 32 % for the 2- (butylamine) ethanethiol radical. For HydroxiPPZ, the substitution was 33 % for the cysteamine group and 66 % for the 1-mercapto-2-propanol radical.

#### 3.1.2 Polymer molecular weight (Mw) determination

Polymer molecular mass was determined by Asymmetric Flow Field Flow Fraction (AF4). As previously described, the Mw of the polymer increases exponentially with the concentration of catalyst used. With a concentration of 2–5 % aluminum chloride (w/w) the Mw obtained is in the 10^4^–10^5^ Da range [14,22]. In this work, we used 7.5 % (w/w) based on the results previously obtained by our group [8].

The molecular mass of the polymer was in the expected range for this catalysed reaction. The number-average molar mass (Mn) for all the polyphosphazenes was within the 5 x 10^4^–10^5^ Da range, while their weight average molar mass (Mw) was between 6 x 10^4^ and 1.5 x 10^5^ kDa (Table 1). These values are far lower than those resulting from other non-catalysed synthesis [23,24], where also Mw/Mn ratio is typically close to 2, indicating broad molecular weight distributions [25]. For the synthesized polyphosphazenes, Mw/Mn values were 1.2–1.4, which is acceptable for biomedical applications [26] (Table 1, Fig. S5).

**Table 1:** Polymer molecular weight and distribution measured by AF4. Mw: Molecular weight; Mn: number-average molar mass.

	Mw (Da)	Mn (Da)	Mw/Mn
AliPPZ	1.48 ± 0.01 x10^5^	1.08 ± 0.1 x10^5^	1.37
2AminePPZ	1.10 ± 0.1 x10^5^	7.98 ± 0.5 x10^4^	1.37
HydroxiPPZ	8.18 ± 0.1 x10^4^	6.50 ± 0.9 x10^4^	1.26
AminePPZ	1.19 ± 0.1 x10^5^	8.83 ± 1.3 x10^4^	1.35
6MHA-PPZ	6.16 ± 0.3 x10^4^	5.11 ± 0.2 x10^4^	1.21

### 3.2 Characterization of polyphosphazene based nanoparticles

#### 3.2.1 Size, zeta potential and concentration of nanoparticles

For the optimization of the nanoparticles, we analyzed how the polymer: plasmid ratio affects their size and surface charge. The ratios are expressed as the molar proportion between cationic PPZ amines (N), anionic PPZ carboxylic acids (C) and DNA phosphates (P).

Cationic nanoparticles were prepared by mixing cationic polyphosphazenes and pDNA at different ratios under mild magnetic stirring. The nanoparticles were characterized for particle size and zeta potential (Table S6). All particles, independent of the cationic polyphosphazene used, showed particle sizes within a small range (95–150 nm), low polydispersity index (PDI <0.2) and positive zeta potential (>+30 mV). It was also observed that both particle size and surface charge increased with a higher N:P ratio (Table S6). The 8:1 N:P ratio allowed to generate NP formulations with zeta potentials close to maximum value, achieved with the 16:1 ratio, but with lower PDI and particle size values. Thus, we selected this 8:1 (N:P) ratio for future experiments.

For the preparation of nanoparticles containing the anionic polyphosphazenes, the plasmid was first mixed with the anionic polymer (6MHA-PPZ) and then added to the cationic polyphosphazene solution under magnetic stirring. The anionic polymer was added at a N:C:P charge ratio of 8:4:1, based on previous published results [8].

The addition of the anionic polymer did not substantially affect the size of the nanoparticles, which was similar when analyzed either by DLS or NTA. Similar polydispersity was observed in the nanoparticles containing 6MHA-PPZ. Regarding the zeta potential, a slight decrease was appreciated for the AliPPZ and 2AminePPZ 8:4:1 prototype (p < 0.001) and did not change for the prototype made with HydroxiPPZ and AminePPZ. Net positive values were maintained in all cases (Table 2).

**Table 2:** Characterization of cationic and cationic/anionic nanoparticles by Dynamic Light Scattering, Laser Doppler Anemometry and Nanoparticle Tracking Analysis. Ps/mL: number of particles/mL.

	**Ratio 8.0.1**				**Ratio 8.4.1**			
	Size (nm)	Zeta Potential (mV)	PDI	Concentration (105 Ps/mL)	Size (nm)	Zeta Potential (mV)	PDI	Concentration (105 Ps/mL)
**AliPPZ**	150 ± 4	38 ± 3	0.2	5.5 ± 0.1	143 ± 4	32 ± 3	0.2	16.9 ± 0.5
**2AminePPZ**	126 ± 4	37 ± 4	0.2	6.9 ± 0.3	129 ± 3	31 ± 3	0.1	15.5 ± 0.4
**HydroxiPPZ**	111 ± 3	38 ± 2	0.1	7.2 ± 0.3	135 ± 3	39 ± 3	0.1	17.1 ± 0.5
**AminePPZ**	119 ± 2	36 ± 2	0.2	5.3 ± 0.2	122 ± 2	35 ± 4	0.2	16.2 ± 0.8

Despite these similarities, the concentration of 8:4:1 NPs was around 3-fold higher than 8:0:1 NPs, as determined by NTA. Besides, nanoparticles containing the anionic PPZ showed a better-defined peak and higher concentration in the mean size (Fig. S7). This suggests that 6MHA-PPZ produces further complexation of cationic PPZ and that such conditions significantly increase polymer incorporation yield. This enhanced incorporation of the cationic polymer leads to a paradoxical outcome: despite the addition of a polyanionic component, the resulting nanoparticles exhibit minimal changes in size and zeta potential. Similar conclusions were drawn in our previous study with AminePPZ and 6MHA-PPZ [8], but herein the tendencies are further extended to the newly synthesized heteropolymers.

#### 3.2.2 Morphological analysis of the nanoparticles

The nanoparticle prototypes were also observed by Field Emission Scanning Electron Microscopy to characterize their morphology and dried particle diameter. This microscopy technique combines scanning transmission electron microscopy and immersion lens detectors. For all formulations the particles were found to be spherical. Nanoparticles having 6MHA-PPZ were more stained, which may be indicative of a higher density compared to the cationic ones. Particle size calculated by FESEM remained in a similar range to that obtained previously by DLS and NTA (Fig. S8).

#### 3.2.3 DNA-binding efficiency of the nanoparticles

**Fig. 4:**
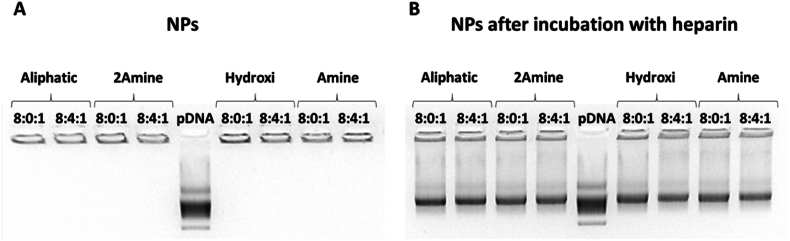
Binding efficiency of the nanosystems determined by a gel retardation assay. a. No bands were observed in the gel corresponding to the nanoparticles, only the band of the free plasmid can be observed. b. Plasmid dissociation after the incubation of the nanoparticles with heparin as anionic competitor.

We also verified the ability of the NPs to bind pDNA, and whether this macromolecule can be dissociated in the presence of a competing polyelectrolyte. This test is necessary to confirm that DNA complexation is not irreversible, and that pDNA might be released intracellularly. In the absence of a competitor, no migration bands were observed for any formulation in a gel electrophoresis test (Fig. 4a), demonstrating that nanoparticles associate completely the plasmid. After the incubation of the NPs with heparin (competing polyelectrolyte), the gel shows migration bands resulting from the dissociation of the plasmid from the polymer (Fig. 4b), which confirms the capacity of the nanoparticles to release the plasmid in intact form.

### 3.3 *In vitro* cytotoxicity of the nanoparticles

#### 3.3.1 Toxicity in a 2D cell model

Once several prototypes with adequate physicochemical properties have been obtained, we tested the cytotoxicity of the formulations starting from 2D cell culture experiments. Specifically, NP cytotoxicity was evaluated by MTS assay using a human glioblastoma cell line (U87MG), 48 h after the addition of the formulations. Concentrations are expressed in μg of plasmid to compare the toxicities of formulations with the same polynucleotide loading. Notably, since all formulations were prepared at the same N:P ratio (8:1), the study was conducted with the same number of cationic groups for the different nanoparticles (Fig. S9). The lethal concentration 50 (LC50) was calculated to compare the cytotoxicity of the different formulations (Table 3), and the values were analyzed statistically.

**Table 3:** Viability determination based on cell metabolic activity of human glioblastoma cell line (U87MG) treated with different concentrations of nanoparticles (ratio 8:0:1 and 8:4:1) at 48 h post-treatment. Data represents the lethal concentration for half of the cell population (LC50). Values are expressed as μg pDNA/cm^2^ and are calculated by extrapolation after the logarithmic representation of the normalized data represented in Fig. S9a. The treatments in statistically homogeneous groups are marked with the same letters (p < 0.001).

Cationic PPZ	Ratio 8:0:1	Ratio 8:4:1
AliPPZ	1.00 ± 0.08^a^	1.46 ± 0.08^a^
2AminePPZ	0.78 ± 0.09 ^b^	1.48 ± 0.15^a^
HydroxiPPZ	0.34 ± 0.02^c^	0.86 ± 0.06 ^b^
AminePPZ	0.74 ± 0.05 ^b^	1.24 ± 0.09^c^

Among the cationic nanoparticles, three distinct toxicity levels were identified (p < 0.001). From least to most toxic, the ranking was: AliPPZ > 2AminePPZ and AminePPZ > HydroxiPPZ. In all cases, the incorporation of 6MHA-PPZ significantly reduced nanoparticle cytotoxicity (p < 0.001). For these cationic/anionic PPZ formulations, three statistically distinct toxicity groups were also observed, with a slightly altered order: AliPPZ and 2AminePPZ > AminePPZ > HydroxiPPZ. This trend largely mirrors the pattern seen in the cationic-only formulations, with minor variations.

Considering this data, we could infer that PPZ with more hydrophobic side groups (i.e. AliPPZ and 2AminePPZ) are generally better tolerated than those with more hydrophilic side groups (i.e. HydroxiPPZ and AminePPZ). Moreover, it is generally accepted that the addition of compatible anionic polymers can improve the cytotoxicity profile of nanoparticles by reducing their charge density [27], an explanation that could fully apply to this study.

#### 3.3.2 Toxicity on a 3D spheroid model

As an intermediate model, between in vitro and in vivo, 3D culture toxicity tests were performed. Only two prototypes with the best transfection/toxicity ratios, AliPPZ and AminePPZ NPs, and their formulations with 6MHA-PPZ, were selected for this phase. The concentration tested in all cases was 2 μg pDNA/mL. The parameters analyzed as indicators of toxicity were: (i) modifications in the size and shape of the neurospheres every 24 h until 72 h, and (ii) the metabolic activity of the neurospheres at 72 h.

**Fig. 5:**
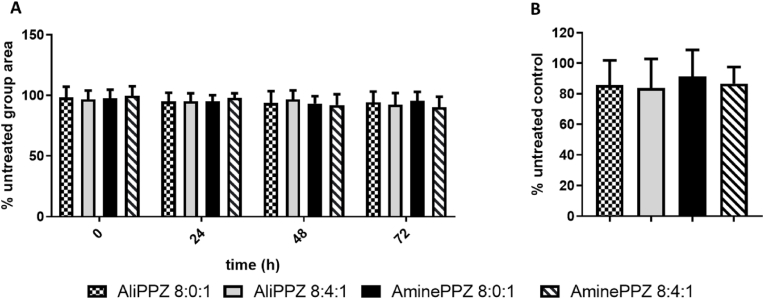
Toxicity in 3D culture models of human glioblastoma cell line (U87MG) after the treatment with the selected formulations at 2 μg pDNA/mL. a. Evolution of the area in the neurospheres treated with the nanoparticles compare with the untreated. b. Alamar Blue assay to determine the metabolic activity 72 h post-treatment compared to the untreated control. (For interpretation of the references to color in this figure legend, the reader is referred to the Web version of this article.)

Similar growth and morphology were observed in all neurospheres, either treated with the negative control or the formulations, indicating a lack of toxicity for the nanoparticles. On the other hand, dissociation and decrease in size was observed in the neurospheres treated with Triton (positive control, Fig. 5a and Fig. S10). The metabolic test also supported the conclusion that the formulations are non-toxic at the tested concentrations (Fig. 5b).

### 3.4 Fish Embryo Acute Toxicity test

The embryos were incubated with five concentrations of the nanoparticles and every 24 h indicators of lethality were observed until the end of the study (96 h). At this point, the LC50 was calculated. The results indicate that AliPPZ nanoparticles are the least toxic (Table 4 and Fig. S11). However, it must be considered that due to their lower amount of charged amines the concentration, the mass of AliPPZ and HydroxiPPZ used doubles that of AminePPZ and 2AminePPZ to maintain an 8:1 charge ratio. When we added the anionic polymer 6MHA-PPZ, the LC50 was improved for all the nanoparticles, in agreement with the tendency found in vitro *(*Figure S9b).

**Table 4:** In vivo toxicity determined by Fish Embryo Acute Toxicity (FET) Test. Results are expressed as ug of cationic polymer/mL of osmosis water required to kill 50 % of the population (LC50).

	AliPPZ	2AminePPZ	HydroxiPPZ	AminePPZ
8:0:1	>10	5.85	5.89	5.52
8:4:1	>10	>10	9.65	9.98

### 3.5 *In vitro* transfection

**Fig. 6:**
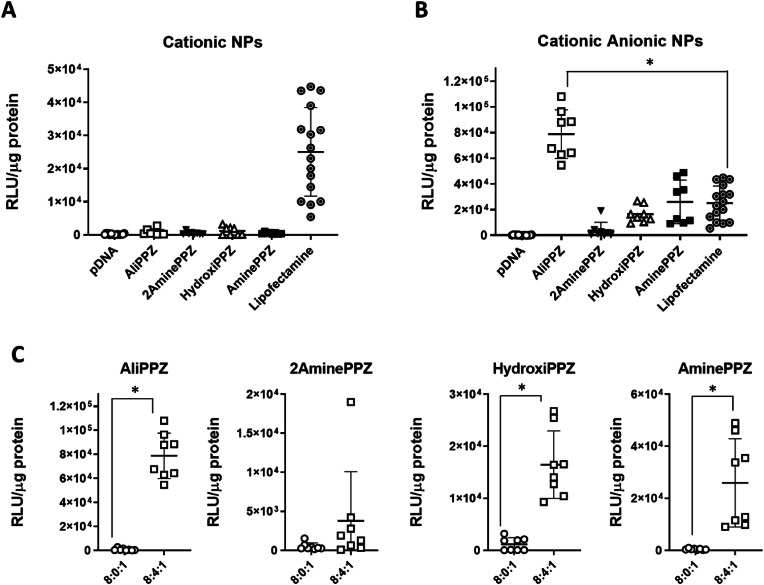
Transfection in bidimensional cultures of human glioblastoma cell line (U87MG) measured 48 h post-transfection by luminescence after the addition of luciferin substrate. Comparison of transfection capacity for the different polymers in the (A) cationic NPs, and (B) the cationic/anionic NPs. (C) Change in transfection levels for each cationic-based nanoparticles after the addition of 6MHA-PPZ. Results are expressed as RLU corrected by quantity of protein. ∗ Statistical analysis at p < 0.001. RLU: relative luminescence units.

The transfection efficiency of the different particles was assessed 48 h post-treatment (Fig. 6 and Fig. S12). It was observed that the transfection of the different cationic nanoparticles was modest and typically well below the benchmark Lipofectamine 2000 (Fig. 6a).

When analysing how the incorporation of 6MHA-PPZ to the nanoparticles affected the cell transfection, we could observe a significant increase (p < 0.001) in all cases, except for the 2Amine prototype (Fig. 6b). Indeed, AminePPZ and HidroxyPPZ nanoparticles had a 100-fold increase when combined with the anionic polymer, equalling their efficacy to Lipofectamine (p < 0.05). For AliPPZ/6MHA-PPZ, NPs cell transfection exceeded by almost 3-fold the levels of Lipofectamine (p < 0.001), and by 300-fold the transfection of the same NP composition without 6MHA-PPZ (Fig. 6c). Furthermore, the differences in transfection could also be observed qualitatively by analyzing GFP expression with fluorescence microscopy (Fig. S12).

### 3.6 Efficacy assay

For the final bioactivity and in vivo tests, the prototype containing a combination of AliPPZ and 6MHAPPZ was selected due to its excellent performance in previous studies. The change of the model plasmid to a therapeutic BMP4 encoding plasmid (pBMP4) did not significantly affect the physicochemical characteristics of the nanoparticles (Fig. S13).

#### 3.6.1 Clonogenicity assay

Herein we investigated the combined effects of pBMP4-nanoparticles and temozolomide (Tz), the first line chemotherapeutic agent used for glioblastoma. This assay was performed to see if this association reduces the clonogenicity of tumor cells compared to the separate treatments [13].

**Fig. 7:**
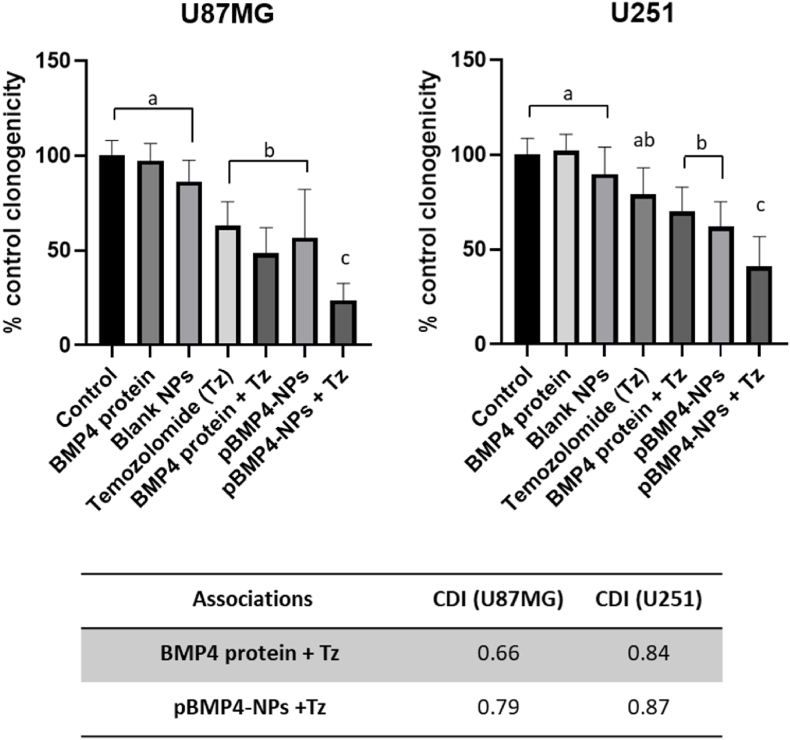
Clonogenicity assay in two glioblastoma cell lines (U251 and U87). Clonogenic capacity of the cells after the incubation during 48h with the different treatments, data are expressed in number of colonies (untreated cells were assumed as 100 %). Table below: Calculation of CDI value to evaluate the combination effect. The treatments in statistically homogeneous groups are marked with the same letters (p < 0.05). Tz: temozolomide; NPs: nanoparticles; BMP4: Bone morphogenic protein-4.

Clonogenicity data showed similar trends for both cell lines (Fig. 7a), but therapeutic effects for BMP4 transfected cells were higher in U87MG [28]. The BMP-4 protein was administered alone as a control and did not reduce the clonogenic capacity of the cells, a result possibly derived from an insufficient dose and protein instability during cell incubation in the culture medium. The pBMP4-loaded NPs showed a reduction in colony formation compared to the blank NPs group, and this effect was comparable to that of the antitumoral drug temozolomide (Tz). The group that combines pBMP4-nanoparticles and Tz showed the highest effect, with clonogenic indexes significantly below those of the treatments given separately.

To determine the effect of the association of the different treatments, their coefficient of drug interaction (CDI) was calculated. Both, the association of BMP4 protein and Tz and the association of pBMP4-NPs and Tz, resulted in CDI <1, indicative of a synergistic effect; however, synergism was higher for the Tz + pBMP4-NPs association (Fig. 7). Moreover, this synergistic association effect was observed both in U87MG and U251.

#### 3.6.2 *In vivo* efficacy

**Fig. 8:**
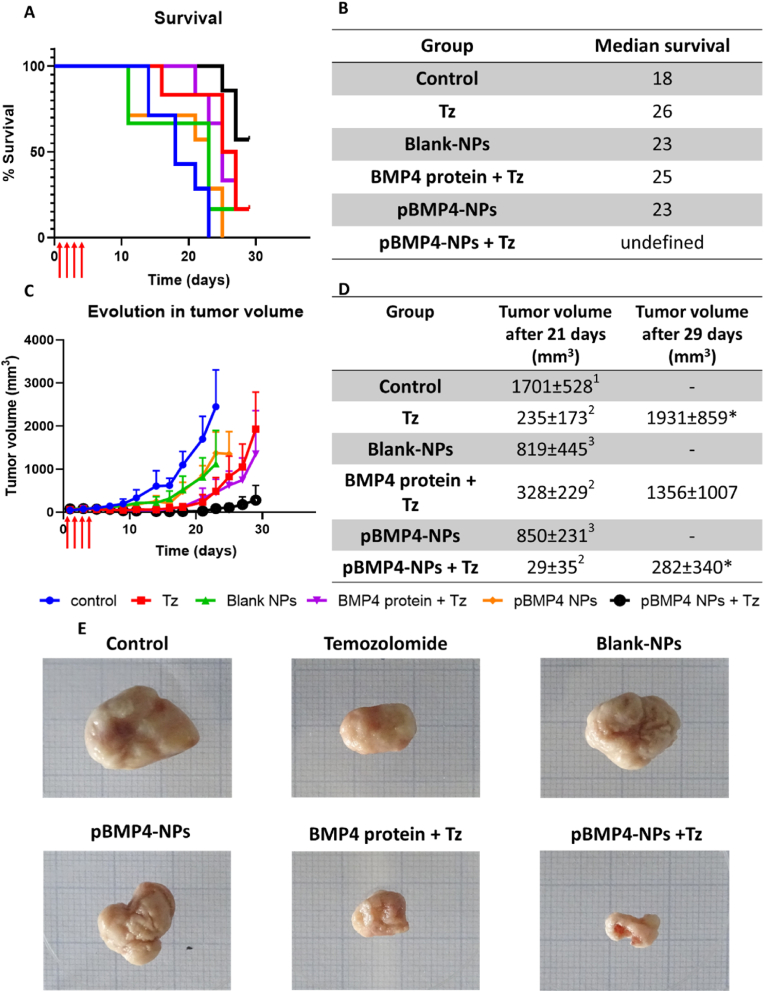
Efficacy in vivo of the formulation alone and in combination with the chemotherapeutic agent, temozolomide. A) Plots of mice survival over time. B) Median survival of the mice after the administration of the different treatments. A Log-rank (Mantel-Cox) test was performed for group comparison (p < 0.001). C) Plots showing the evolution of tumor volume for each group. D) Key parameters related to tumor evolution, final volume at 21 days and 29 days. A one-way ANOVA with Turkey's multiple comparisons test was used for group comparison at 21 days (p < 0.0001) and 29 days (p < 0.05). Statistically homogeneous groups are indicated by the same numerical superscript next to the 21-day value. On the day 29 groups that are statistically different between them are marked with an asterisk (∗). E) Images of the tumors after euthanasia. Animals with tumor necrosis or a size greater than 1500 mm^3^, were sacrificed to reduce the suffering of the animal. The red arrows illustrate the four administrations on consecutive days. Tz: Temozolomide, NPs: Nanoparticles; BMP4: Bone morphogenic protein-4. (For interpretation of the references to color in this figure legend, the reader is referred to the Web version of this article.)

We tested the efficacy of pBMP4-NPs in a U87MG xenograft mice model. After administration of the different treatments, tumor size was followed for one month. Given that there is always some heterogenicity at the beginning in the tumor size, the mice were distributed to keep the groups comparable. Besides, the results were also corrected respect to the initial tumor volume (Fig. S14), although there were no important changes in relative trends between the groups whether analyzing corrected or non-corrected tumor volume (see Fig. 8c vs Fig. S14a).

No variations in mice weight were observed during the experiment, indicating no signs of toxicity. On the other hand, there were clear differences in animal survival, where a clear increase was observed for the group treated with the Tz + pBMP4-NPs association (Fig. 8a). Median survival was calculated to discern the differences among groups. The control group had the lowest survival (18 days), while all monotherapies and the BMP4 protein + Tz group had intermediate survivals (23–26 days). The group treated with pBMP4-NPs + Tz showed a higher median survival (above 50 %) by the end of the assay (30 days, Fig. 8b). The survival curves differed significantly according to a Log-rank (Mantel-Cox) test (p < 0.001).

To analyze this data in more detail, tumor volume was also analyzed. The data showed that Blank NPs and pBMP4-NPs showed a very slight tumor size reduction, while higher tumor reduction was observed when the animals were treated with Tz or with pBMP4-NPs. The largest reduction was achieved with the association of Tz and pBMP4-NPs, where the tumor was almost in stasis (Fig. 8c and d). This enhanced efficacy of the association of a cytostatic and a gene therapy for BMP4 agreed with the previous in vitro data and with the survival curves.

Further statistical analysis was performed by comparing the tumor volume at defined points by a one-way Anova and post-hoc Tuckey's multiple comparison test. A first analysis was performed at 21 days, the last time-point where all experimental groups can be analyzed. Tumor volume at 21 days showed three statistically homogenous groups (p < 0.0001): (1) Control, (2) Control NPs and pBMP4 NPs, and (3) Tz, Tz + BMP4 protein, and Tz + pBMP4 NPs (Fig. 8d). A second statistical comparison was performed at day 29 with the three remaining groups (Tz, Tz + BMP4 protein, and Tz + pBMP4 NPs). In this case statistical differences were found between Tz and the Tz + pBMP4 NPs (p < 0.05).

After sacrificing the animals, the tumors were extracted for imaging and morphological analysis (Fig. 8e), and for further processing for PCR. When considering differences in tumor volume, it is important to bear in mind that the animals from the control group were sacrificed by day 23 or before, due to tumor burden. Some of the mice from the Blank NPs and the pBMP4-NPs were also euthanized before day 25 due to necrosis at the injection site. Still, the morphologies and size observed clearly indicate an antitumoral process in both groups treated with the combination Tz + BMP4.

**Fig. 9:**
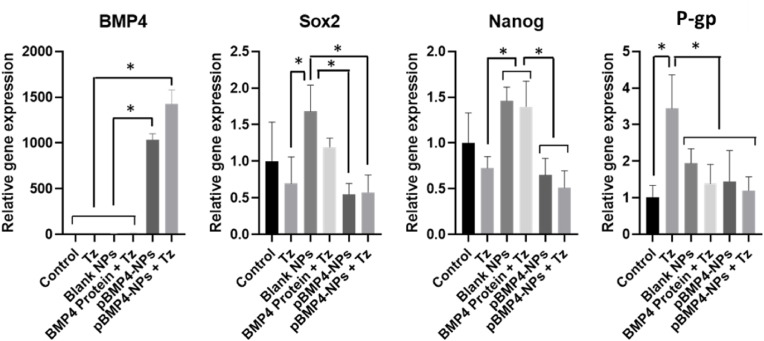
RNA expression of CSCs markers and efflux pumps after the administration of the different treatments in a glioblastoma xenograft model, measured by real time-PCR. Other genes were also studied, but no differences were observed between the different treatments (Figure S4.2). ∗Statistical analysis at p < 0.05.

To further elucidate the mechanisms underlying the observed therapeutic effects, we performed real-time PCR analysis on tumor samples collected at the study endpoint. Notably, BMP4 expression was over 1000-fold higher in tumors treated with pBMP4-NPs, confirming successful and sustained transgene delivery. None of the other groups showed intrinsic BMP4 signalling (Fig. 9). This result indicates the capacity of our NPs to generate sustained expression of the transgene at the injection site, even 25 days after the last injection.

Nestin [29], Nanog [30], Oct4 [31] and Sox2 [32] are genes implicated in the CSCs properties and are overexpressed in glioblastoma [29,33]. We have shown in previous studies with primary glioblastoma models that BMP signalling can reduce the expression of these markers. However, there was not a very clear tendency in the data besides some small changes reaching statistical significance (Fig. 9).

We also analyzed the expression of P-glycoprotein (P-gp) a drug efflux pump located on CSCs membrane that confers multidrug resistance by active transport of drugs and is involved in chemotherapy resistance [34]. We observed that after the administration of Tz, the expression of this gene was upregulated by 3-fold as compared to control cells. The association of Tz with BMP4 protein or pBMP4-NPs normalized P-gp expression to basal levels. This normalization might be the subjacent molecular mechanism behind the synergistic combination of Tz and pBMP4-NPs.

## 4 Discussion

Due to their capacity to accommodate a great variety of chemical functionalities, polyphosphazenes (PPZs) have been proposed as cutting-edge materials for gene delivery [4]. In this work, we analyze cationic polyphosphazenes to determine which compositions yield the best gene delivery results, particularly in combination with 6MHA-PPZ, an anionic polymer with transfection enhancing characteristics [8]. The modifications tested in this study were other chemical functionalities, to be introduced together with a primary amine group. These modifications led to heteropolymers that included secondary amine groups (2AminePPZ), non-ionic hydrophilic (hydroxyl, HydroxiPPZ) groups, and hydrophobic groups (AliPPZ). Together with these materials we also tested as reference a cationic homopolymer (AminePPZ), previously used for gene delivery (Fig. 1) [8].

All synthesized polyphosphazenes demonstrated the capacity to form nanoparticles upon complexation with DNA, underscoring their inherent versatility for gene delivery applications. The incorporation of 6MHA-PPZ into cationic PPZ-pDNA complexes led to a notable increase in nanoparticle yield, while having minimal impact on particle size or zeta potential—suggesting the formation of more compact structures. These findings are consistent with previous observations [8]. All cationic polyphosphazenes exhibited comparable cytotoxicity profiles and similar lethal dose values in zebrafish models. Importantly, the inclusion of 6MHA-PPZ consistently enhanced the biocompatibility of the formulations. As reported for other polymer systems [35], this improvement may stem from the ability of 6MHA-PPZ to modulate the spatial distribution of surface charges on the nanoparticles. However, it is important to note that zeta potential measurements reflect only the net surface charge and do not capture charge heterogeneity. Additionally, 6MHA-PPZ may contribute to improved colloidal stability and steric shielding, thereby reducing nonspecific cellular interactions and membrane disruption [35].

When analyzing the gene delivery characteristics of nanoparticles based on pure cationic polyphosphazenes, all systems performed similarly, achieving moderate transfection levels. However, as observed before for AminePPZ [8], these levels improved significantly when 6MHA-PPZ was incorporated into the system. An exception was the case of nanoparticles based on 2AminePPZ, which did not improve their transfection with the anionic polymer. We hypothesize that nanoparticles based on 2AminePPZ may already possess good endosomal escape capacity and that their moderate gene transport activity might be due to other deficiencies that the incorporation of the anionic polymer cannot address.

Conversely, AliPPZ nanoparticles showed the most significant improvement with the incorporation of the anionic PPZ, achieving high expression levels, surpassing those of the benchmark used in the study. This result is particularly promising as it suggests that hydrophobicity can enhance transfection efficiency. Hydrophobic modifications may facilitate better interaction with cell membranes or greater stability of the nanoparticles in the biological environment, resulting in higher efficiency in delivering genetic material. Similar effects of hydrophobic modifications have been observed for PEI [36] and other polymers [37]. Based on these considerations, the prototype combining the anionic polymer 6MHA-PPZ and the hydrophobic polymer AliPPZ was selected for the delivery of a therapeutic gene.

BMPs are proteins with the capacity to supress glioblastoma initiating cells. We hypothesize that their therapeutic efficacy in vivo may be limited unless they are delivered via an appropriate carrier that can enhance their biological half-life and target the brain region effectively. Initial tests with BMP7-loaded microspheres demonstrated antitumoral effects in glioblastoma models both in vitro and in vivo [38,39]. In this study, we aim to use gene therapy to achieve sustained levels of BMP4 in tumors.

Our research has shown that nanoparticles effectively deliver the therapeutic gene pBMP4 both in cell cultures and in vivo. Notably, BMP4 was overexpressed by more than 1000-fold in tumors treated with pBMP4-NPs as compared to those of the control, even 25 days after the last injection. This suggests that this approach could be effective for therapies that modulate the tumor microenvironment [1].

Combining pBMP4-NPs with the chemotherapeutic agent Tz resulted in a significant reduction in clonogenic capacity and tumor volume compared to control groups or separate therapies. Mathematical analysis indicated a clear synergistic effect of the BMP4 + Tz combination, and even more so for the pBMP4-NP + Tz combination in vitro. In vivo, the pBMP4 NP + Tz combination significantly reduced tumor growth compared to other treatments or controls, with more than 50 % of animals surviving 30 days post-tumor implantation.

The mechanism behind the antitumoral effect of pBMP4 NPs + Tz remains unclear. Previous studies suggest that BMP4 works through the directed differentiation of glioblastoma initiating cells [9,[40], [41], [42]]. However, our studies showed limited effects of BMP4 in protein form, and its impact on gliobastoma initiating cell-related gene expression (Nestin, Nanog, Oct4, and Sox2) after pBMP4-NP treatment remains inconclusive. This may be due to the limitations of the U87MG tumor model, which does not clearly exhibit a CSC phenotype, as U87MG cells have less than 1 % CSC population under standard culture conditions [[43], [44], [45]]. Conversely, about 50 % of primary glioblastomas are known to be sensitive to BMP signalling [41]. Still, this study highlights the potential of combining glioblastoma initiating cell-specific therapies with conventional cytostatic drugs for glioblastoma treatment, as discussed in previous studies [1,46,47].

Although the effect on the glioblastoma initiating cell signature was unclear, pBMP4 had a therapeutic impact beyond antitumoral effects, particularly in the expression of the efflux pump P-gp. Tz treatment caused an up regulation of this gene, a typical cellular defence mechanism. However, when pBMP4-NPs were used in combination, the expression levels of this gene were normalized. This effect on efflux pumps has been noted in other nanomedicine studies [[48], [49], [50]], and could explain the observed synergistic effects in vitro and in vivo. Considering the data, we suggest that this combination gene therapy could have significant clinical potential for treating glioblastoma, especially in stratified, BMP-sensitive tumors.

Finally, it could be of interest to investigate the synergistic effect of combining these BMP-loaded nanoparticles with innovative physical therapies such as photodynamic therapy, tumor-treating field therapy, and cold atmospheric plasma therapy [51].

## 5 Conclusion

This study presents a significant advancement in the design of polyphosphazene-based gene delivery systems. We synthesized and characterized three novel cationic heteropolyphosphazenes—AliPPZ, 2AminePPZ, and HydroxiPPZ—for the first time, and systematically evaluated their performance in gene delivery. Among them, AliPPZ, a hydrophobically modified polymer, demonstrated superior transfection efficiency, outperforming both commercial benchmarks and previously reported prototypes.

The incorporation of the anionic polymer 6MHA-PPZ consistently enhanced the safety and transfection efficiency of all tested formulations, with the AliPPZ/6MHA-PPZ combination emerging as the most effective. This highlights the potential of combining cationic/hydrophobic and anionic polymers to optimize gene delivery platforms.

From a therapeutic perspective, this work pioneers the use of polyphosphazene-based nanoparticles for the delivery of BMP4-encoding plasmids in glioblastoma models. The optimized pBMP4-NPs, particularly when combined with temozolomide, significantly reduced tumor growth and improved survival in preclinical models. These findings suggest a promising and synergistic strategy for targeting glioblastoma, especially in BMP-sensitive tumors, and open new avenues for gene therapy in oncology.

## CRediT authorship contribution statement

**Carla Garcia-Mazas:** Writing – original draft, Visualization, Project administration, Methodology, Investigation, Formal analysis, Data curation, Conceptualization. **Elia Bozzato:** Methodology, Investigation, Formal analysis, Data curation. **Jhonathan Angel Araujo Fernandez:** Writing – review & editing, Methodology, Investigation, Formal analysis, Data curation. **Federico Quattrini:** Writing – review & editing, Methodology, Investigation, Formal analysis, Data curation. **Veronique Preat:** Writing – review & editing, Supervision, Project administration, Methodology. **Laura Sanchez:** Writing – review & editing, Validation, Supervision, Methodology. **Noemi Csaba:** Writing – review & editing, Supervision, Project administration, Methodology, Investigation. **Marcos Garcia-Fuentes:** Writing – review & editing, Writing – original draft, Validation, Supervision, Resources, Project administration, Methodology, Funding acquisition, Formal analysis, Data curation, Conceptualization.

## Declaration of competing interest

The authors declare the following financial interests/personal relationships which may be considered as potential competing interests: Marcos Garcia-Fuentes reports financial support provided by 10.13039/501100004837Ministerio de Ciencia e Innovación (Spain) and 10.13039/501100004587Instituto de Salud Carlos III. Carla Garcia-Mazas reports financial support provided by el Ministerio de Education, Cultura y Deportes (Spain). Marcos Garcia-Fuentes, Noemi Csaba and Carla García-Mazas have patent #EP4239013A1 issued to University of Santiago de Compostela. These authors declare no other conflicts of interest. Other authors declare that they have not known competing financial interests or personal relationships that could have appeared to influence the work reported in this paper.

## Data Availability

Data will be made available on request.

## References

[1] Garcia-Mazas C., Csaba N., Garcia-Fuentes M. (2017). Biomaterials to suppress cancer stem cells and disrupt their tumoral niche. Int. J. Pharm..

[2] Xing H., Lu M., Yang T., Liu H., Sun Y., Zhao X., Xu H., Yang L., Ding P. (2019). Structure-function relationships of nonviral gene vectors: lessons from antimicrobial polymers. Acta Biomater..

[3] Beach M.A., Nayanathara U., Gao Y., Zhang C., Xiong Y., Wang Y., Such G.K. (2024). Polymeric nanoparticles for drug delivery. Chem. Rev..

[4] Hsu W.H., Csaba N., Alexander C., Garcia-Fuentes M. (2020). Polyphosphazenes for the delivery of biopharmaceuticals. J. Appl. Polym. Sci..

[5] De Wolf H.K., De Raad M., Snel C., Van Steenbergen M.J., Fens M.H.A.M., Storm G., Hennink W.E. (2007). Biodegradable poly(2-dimethylamino ethylamino)phosphazene for in vivo gene delivery to tumor cells. Effect of polymer molecular weight. Pharm. Res..

[6] Yang Y., Zhang Z., Chen L., Gu W., Li Y. (2010). Galactosylated poly(2-(2-aminoethyoxy)ethoxy)phosphazene/DNA complex nanoparticles: in vitro and in vivo evaluation for gene delivery. Biomacromolecules.

[7] Ma C., Zhang X., Du C., Zhao B., He C., Li C., Qiao R. (2016). Water-soluble cationic polyphosphazenes grafted with cyclic polyamine and imidazole as an effective gene delivery vector. Bioconjug. Chem..

[8] Hsu W.-H., Sánchez-Gómez P., Gomez-Ibarlucea E., Ivanov D.P., Rahman R., Grabowska A.M., Csaba N., Alexander C., Garcia-Fuentes M. (2019). Structure-optimized interpolymer polyphosphazene complexes for effective gene delivery against glioblastoma. Adv. Ther..

[9] Piccirillo S.G.M., a Reynolds B., Zanetti N., Lamorte G., Binda E., Broggi G., Brem H., Olivi a, Dimeco F., Vescovi a L. (2006). Bone morphogenetic proteins inhibit the tumorigenic potential of human brain tumour-initiating cells. Nature.

[10] Westerfield M. (2007).

[11] OECD (2013). Test No. 236: fish embryo acute toxicity (FET) test.

[12] Kimmel C.B., Ballard W.W., Kimmel S.R., Ullmann B., Schilling T.F. (1995). Stages of embryonic development of the zebrafish. Dev. Dyn..

[13] Zhao M., Bozzato E., Joudiou N., Ghiassinejad S., Danhier F., Gallez B., Préat V. (2019). Codelivery of paclitaxel and temozolomide through a photopolymerizable hydrogel prevents glioblastoma recurrence after surgical resection. J. Contr. Release.

[14] Sohn Y.S., Cho Y.H., Baek H., Jung O.S. (1995). Synthesis and properties of low molecular weight polyphosphazenes. Macromolecules.

[15] Allcock H.R., Kugel R.L. (1965). Synthesis of high polymeric alkoxy-and aryloxyphosphonitriles. J. Am. Chem. Soc..

[16] Allcock H.R. (2017). Encyclopedia of Inorganic and Bioinorganic Chemistry.

[17] Hagnauer G.L. (1981). Polydichlorophosphazene polymerization studies. J. Macromol. Sci. Part A - Chemistry.

[18] Gimblett F.G.R. (1960). Catalysts for the bulk polymerization of phosphonitrilic chloride trimer: I. Benzoic acid. Polymer (Guildf.).

[19] Allcock H.R., Kugel R.L., Compounds Phosphonitrilic (1966). VII. High molecular weight poly(diaminophosphazenes). Inorg. Chem..

[20] Borisov A.S., Hazendonk P., Hayes P.G. (2010). 31P MAS NMR spectroscopy of hexachlorocyclotriphosphazene at different stages during thermal ring-opening polymerization. J. Inorg. Organomet. Polym. Mater..

[21] Henke H., Wilfert S., Iturmendi A., Brüggemann O., Teasdale I. (2013). Branched polyphosphazenes with controlled dimensions. J. Polym. Sci. Polym. Chem..

[22] Allcock H.R., Crane C.A., Morrissey C.T., Nelson J.M., Reeves S.D., Honetman C.H., Manners I. (1996). “Living” cationic polymerization of phosphoranimines as an ambient temperature route to polyphosphazenes with controlled weights. Macromolecules.

[23] Singler R.E., Schneider N.S., Hagnauer G.L. (1975). Polyphosphazenes: synthesis—properties—applications. Polym. Eng. Sci..

[24] Andrianov A.K., Chen J., LeGolvan M.P. (2004). Poly(dichlorophosphazene) as a precursor for biologically active polyphosphazenes: synthesis, characterization, and stabilization. Macromolecules.

[25] Shrivastava A. (2018). Introduction to plastics engineering.

[26] Quiñones J.P., Roschger C., Iturmendi A., Henke H., Zierer A., Peniche-Covas C., Brüggemann O. (2022). Polyphosphazene-based nanocarriers for the release of camptothecin and epirubicin. Pharmaceutics.

[27] Wong S.Y., Pelet J.M., Putnam D. (2007). Polymer systems for gene delivery: past, present, and future. Prog. Polym. Sci..

[28] Zhang Y., Wen Y.L., Ma J.W., Ye J.C., Wang X., Huang J.X., Meng C.Y., Xu X.Z., Wang S.X., Zhong X.Y. (2017). Tetrandrine inhibits glioma stem-like cells by repressing β-catenin expression. Int. J. Oncol..

[29] Zhang M., Song T., Yang L., Chen R., Wu L., Yang Z., Fang J. (2008). Nestin and CD133: valuable stem cell-specific markers for determining clinical outcome of glioma patients. J. Exp. Clin. Cancer Res..

[30] Moon J.H., Kwon S., Jun E.K., Kim A., Whang K.Y., Kim H., Oh S., Yoon B.S., You S. (2011). Nanog-induced dedifferentiation of p53-deficient mouse astrocytes into brain cancer stem-like cells. Biochem. Biophys. Res. Commun..

[31] Du Z., Jia D., Liu S., Wang F., Li G., Zhang Y., Cao X., Ling E.A., Hao A. (2009). Oct4 in expressed in human gliomas and promotes colony formation in glioma cells. Glia.

[32] Jia X., Li X., Xu Y., Zhang S., Mou W., Liu Y., Liu Y., Lv D., Liu C.H., Tan X., Xiang R., Li N. (2011). SOX2 promotes tumorigenesis and increases the anti-apoptotic property of human prostate cancer cell. J. Mol. Cell Biol..

[33] Guo Y., Liu S., Wang P., Zhao S., Wang F., Bing L., Zhang Y., Ling E.A., Gao J., Hao A. (2011). Expression profile of embryonic stem cell-associated genes Oct4, Sox2 and nanog in human gliomas. Histopathology.

[34] Nakai E., Park K., Yawata T., Chihara T., Kumazawa A., Nakabayashi H., Shimizu K. (2009). Enhanced mdr1 expression and chemoresistance of cancer stem cells derived from glioblastoma. Cancer Invest..

[35] Richter F., Leer K., Martin L., Mapfumo P., Solomun J.I., Kuchenbrod M.T., Hoeppener S., Brendel J.C., Traeger A. (2021). The impact of anionic polymers on gene delivery: how composition and assembly help evading the toxicity-efficiency dilemma. J. Nanobiotechnol..

[36] Teo P.Y., Yang C., Hedrick J.L., Engler A.C., Coady D.J., Ghaem-Maghami S., George A.J.T., Yang Y.Y. (2013). Hydrophobic modification of low molecular weight polyethylenimine for improved gene transfection. Biomaterials.

[37] Pearce A.K., O'Reilly R.K. (2021). Polymers for biomedical applications: the importance of hydrophobicity in directing biological interactions and application efficacy. Biomacromolecules.

[38] Reguera-Nuñez E., Roca C., Hardy E., de la Fuente M., Csaba N., Garcia-Fuentes M. (2014). Implantable controlled release devices for BMP-7 delivery and suppression of glioblastoma initiating cells. Biomaterials.

[39] Crecente-Campo J., Borrajo E., Vidal A., Garcia-Fuentes M. (2017). New scaffolds encapsulating TGF-β3/BMP-7 combinations driving strong chondrogenic differentiation.

[40] Bai F., Wang C., Lu Q., Zhao M., Ban F.Q., Yu D.H., Guan Y.Y., Luan X., Liu Y.R., Chen H.Z., Fang C. (2013). Nanoparticle-mediated drug delivery to tumor neovasculature to combat P-gp expressing multidrug resistant cancer. Biomaterials.

[41] Gonzalez-Gomez P., Anselmo N.P., Mira H. (2014). BMPs as therapeutic targets and biomarkers in astrocytic glioma. BioMed Res. Int..

[42] Liu S., Yin F., Zhao M., Zhou C., Ren J., Huang Q., Zhao Z., Mitra R., Fan W., Fan M. (2016). The homing and inhibiting effects of hNSCs-BMP4 on human glioma stem cells. Oncotarget.

[43] Hirschmann-Jax C., Foster A.E., Wulf G.G., Nuchtern J.G., Jax T.W., Gobel U., Goodell M.A., Brenner M.K. (2004). A distinct “side population” of cells with high drug efflux capacity in human tumor cells. Proc. Natl. Acad. Sci. U. S. A..

[44] cang Yu S., fang Ping Y., Yi L., hua Zhou Z., hong Chen J., hong Yao X., Gao L., Wang J.M., wu Bian X. (2008). Isolation and characterization of cancer stem cells from a human glioblastoma cell line U87. Cancer Lett..

[45] Balça-Silva J., Matias D., Dubois L.G., Carneiro B., do Carmo A., Girão H., Ferreira F., Ferrer V.P., Chimelli L., Filho P.N., Tão H., Rebelo O., Barbosa M., Sarmento-Ribeiro A.B., Lopes M.C., Moura-Neto V. (2017). The expression of connexins and SOX2 reflects the plasticity of glioma stem-like cells. Transl. Oncol..

[46] Sai K., Li W.-Y., Chen Y.-S., Wang J., Guan S., Yang Q.-Y., Guo C.-C., Mou Y.-G., Li W.-P., Chen Z.-P. (2014). Triptolide synergistically enhances temozolomide-induced apoptosis and potentiates inhibition of NF-κB signaling in glioma initiating cells. Am. J. Chin. Med..

[47] Bausart M., Préat V., Malfanti A. (2022). Immunotherapy for glioblastoma: the promise of combination strategies. J. Exp. Clin. Cancer Res..

[48] Vinogradov S., Wei X. (2012). Cancer stem cells and drug resistance: the potential of nanomedicine. Nanomedicine.

[49] Ortíz R., Quiñonero F., García-Pinel B., Fuel M., Mesas C., Cabeza L., Melguizo C., Prados J. (2021). Nanomedicine to overcome multidrug resistance mechanisms in Colon and pancreatic cancer: recent progress. Cancers (Basel).

[50] Halder J., Pradhan D., Kar B., Ghosh G., Rath G. (2022). Nanotherapeutics approaches to overcome P-glycoprotein-mediated multi-drug resistance in cancer. Nanomedicine.

[51] Bai F., Deng Y., Li L., Lv M., Razzokov J., Xu Q., Xu Z., Chen Z., Chen G., Chen Z. (2024). Advancements and challenges in brain cancer therapeutics. Exploration.

