# Supplementary material for: Designing polyphosphazene derivatives for gene delivery in glioblastoma treatment
Source: Mater Today Bio. 2025 Jun 25;33:102010. doi: 10.1016/j.mtbio.2025.102010 (PMC12271426; doi:10.1016/j.mtbio.2025.102010)

S1. Plasmid amplification

To perform the optimization of the nanoparticles we used a plasmid (pEGFPLuc), which expresses two reporter proteins: enhanced Green Fluorescent Protein and Luciferase. To perform the nanoparticle efficacy tests, we selected a plasmid encoding Bone Morphogenic Protein-4 (pBMP4).


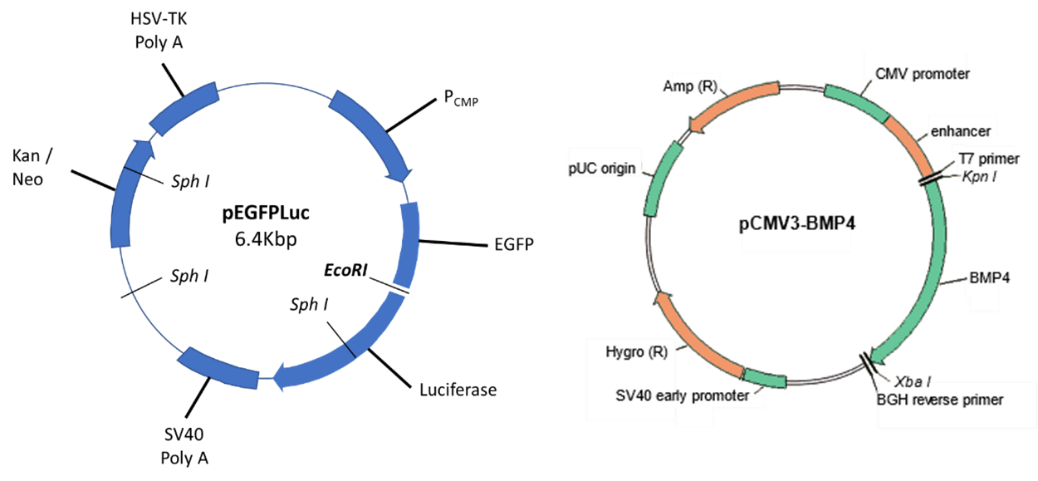


Figure S1. EGFP-Luc and BMP4 plasmid map.

Competent E. coli DH5α bacteria were transformed with pEGFPLuc or pBMP4, resuspended in Luria-Bertani (LB) medium and incubated for 2 h at 37 °C with orbital agitation. Bacteria were selected through seeding on Petri dishes and supplementation of the media with the selection antibiotic (10μg/mL Kanamycin in the case of pEGFP-Luc and 50 μg ampicillin / mL in the case of pBMP4). The bacteria were incubated in this selection media at 37 °C for 24 h. An isolated colony was grown in supplemented LB in the orbital incubator and amplified until reaching an adequate number of bacteria to perform plasmid extraction.

Plasmid extraction was performed with an Invitrogen™ PureLink™ HiPure Plasmid Gigaprep Kit (Thermo Fisher, USA) following the manufacturer’s instructions. Briefly, bacteria were centrifuged at 5000 rcf for 15 min, the pellet was resuspended in a RNase solution to remove the RNA and mixed with a lysis buffer to release the intracellular content. In order to remove the cellular debris, a precipitation buffer was added to the solution and filtered. Then, the pDNA was purified using the by columns provided in the kit. Finally, the plasmid was precipitated, washed, and quantified by UV absorption (Nanodrop, ThermoFisher, USA).

S2. Scheme of the zebrafish embryo acute toxicity test procedure.

**
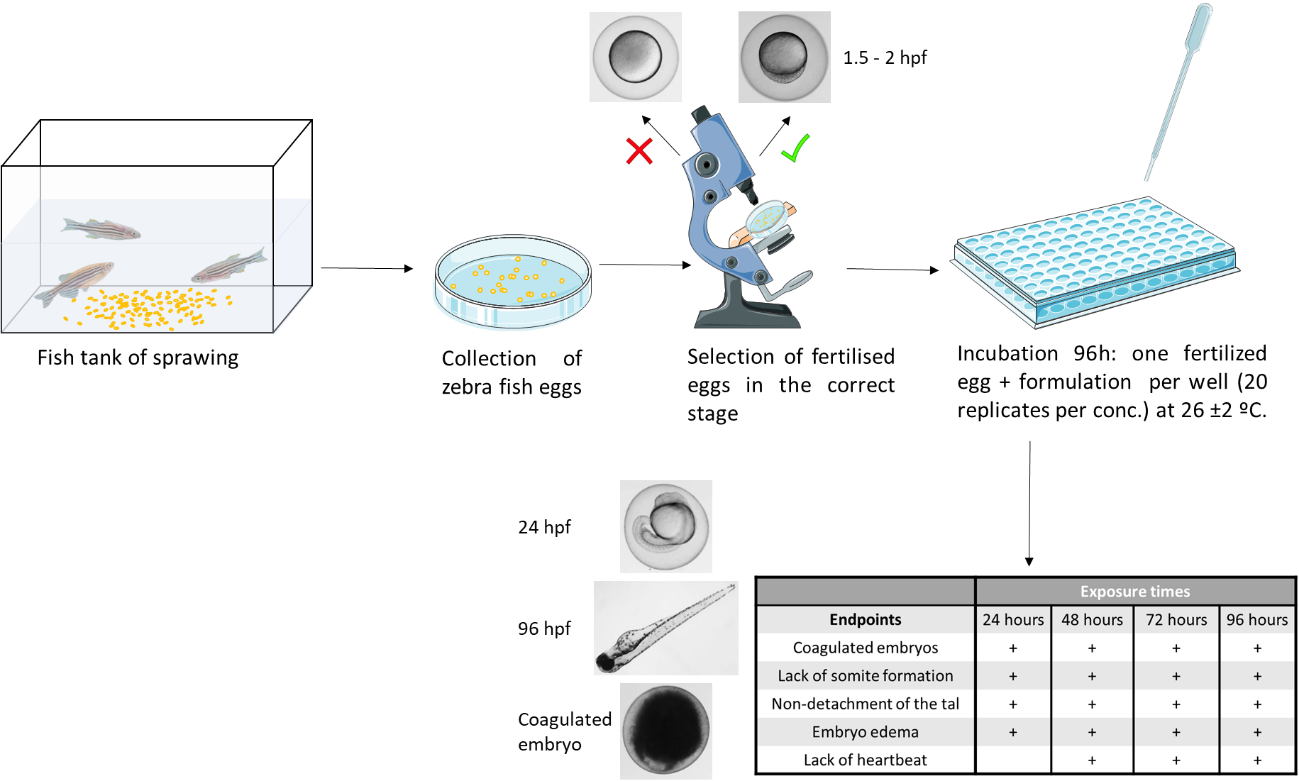
**

S3. Table of primer sequences and thermal cycler conditions

Lyophilized primers obtained from ThermoFisher were resuspended in DNAse / RNAse water to a stock solution of 100 μM. The thermal cycler conditions were as follows: 95 °C for 2 minutes, followed by 40 cycles of 95 °C for 15 seconds, 55 °C for 10 seconds, and 68 °C for 20 seconds.

|  | **Forward** | **Reverse** |
| --- | --- | --- |
| **GAPDH** | 5´-GCCAAGGTCATCCATGACAACT-3´ | 5´-AGGGCCATCCACAGTCTTCTG-3´ |
| **Sox2** | 5´-ACACCAATCCCATCCACACT-3´ | 5´-GCAAACTTCCTGCAAAGCTC-3´ |
| **Nestin** | 5´-CCTCCTGGAGGCTGAGAACTC-3´ | 5´-AAGGCTGGCACAGGTGTCTC-3´ |
| **Oct4** | 5´-CCCGCCGTATGAGTTCTGTGG-3´ | 5´-CCGGGTTTTGCTCCAGCTTCTC-3´ |
| **Nanog** | 5´-CCGCGCCCTGCCTAGAAAAGAC-3´ | 5´-AGCCTCCCAATCCCAAACAATACG-3´ |
| **BMP4** | 5´-AATGTGACACGGTGGGAAACT-3´ | 5´-CCCGCTGTGAGTGATGCTTA-3´ |
| **MDR1** | 5´-AACAACGCATTGCCATAGCTCGTG-3´ | 5´-AGTCTGCATTCTGGATGGTGGACA-3´ |
| **MRP1** | 5´-CATCGTTCTGTTTGCTGCCCTGTT-3´ | 5´-AGTACGTGGTGACCTGCAATGAGT-3´ |
| **ABCG2** | 5´-GCCACGTGATTCTTCCACAA-3´ | 5´-TTCTGCCCAGGACTCAATGC-3´ |

S4. Polymer characterization by NMR

1. AAPPZ Characterization

^31^P-NMR


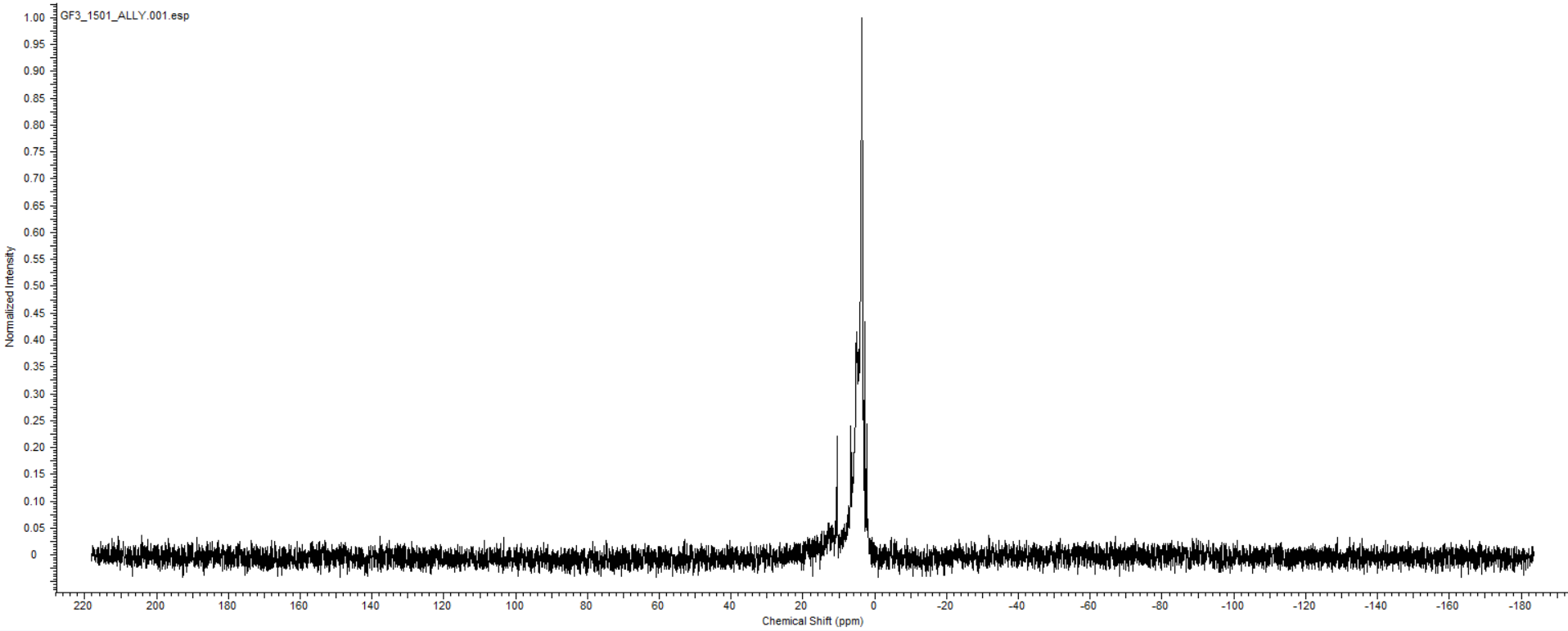


^1^H-NMR and DOSY spectrum


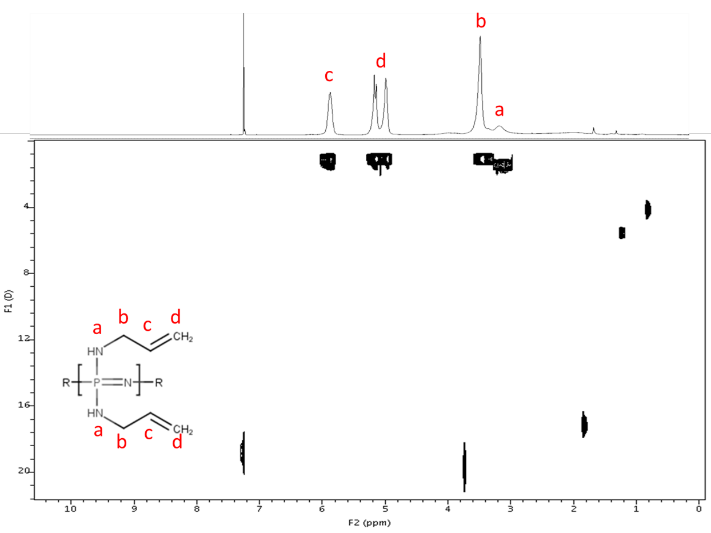


| Polymer | ^31^P-NMR Peak (ppm) |
| --- | --- |
| Allylamine precursor (AAPPZ) | 3.64 |
| Aliphatic-PPZ | 4.44 |
| 2Amine-PPZ | 4.94 |
| Hydroxi-PPZ | 4.95 |
| Amine-PPZ | 4.88 |
| 6MHA-PPZ | 4.86 |

1. ^31^P-NMR characterization
2. Majority and minority substitution of AAPPZ.

Depending on which of the AAPPZ-vinyl carbons is attacked during the addiction reaction, two possible substitutions can be generated. Therefore, the product results in both a majority form (≈90% addiction at the terminal carbon) and a minority form (≈10% addiction at the intermediate carbon). ^1^H-NMR spectra of the polymers show three common peaks to all the modified polymers (a, b and c; Fig. 5) typical of the substituted allylamine in the majority form. A small peak at 1.4 ppm correspond to group c’ in the minority form.

**
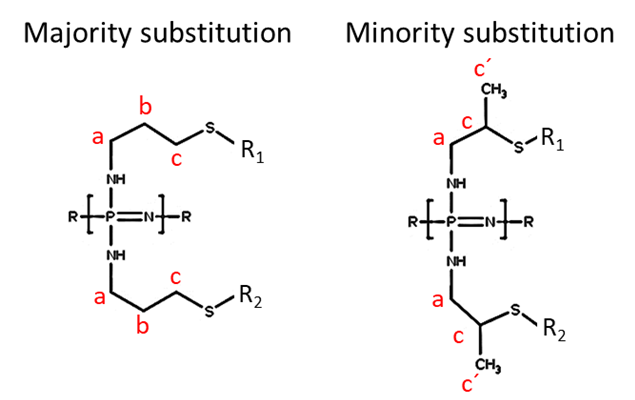
**

1. **Bidimensional NMR: COSY and HSQC.** Comparison of bidimensional-NMR spectra of the different polymers. (a) ^1^H-^1^H COSY spectra and (b) ^1^H-^13^C HSQC spectra.


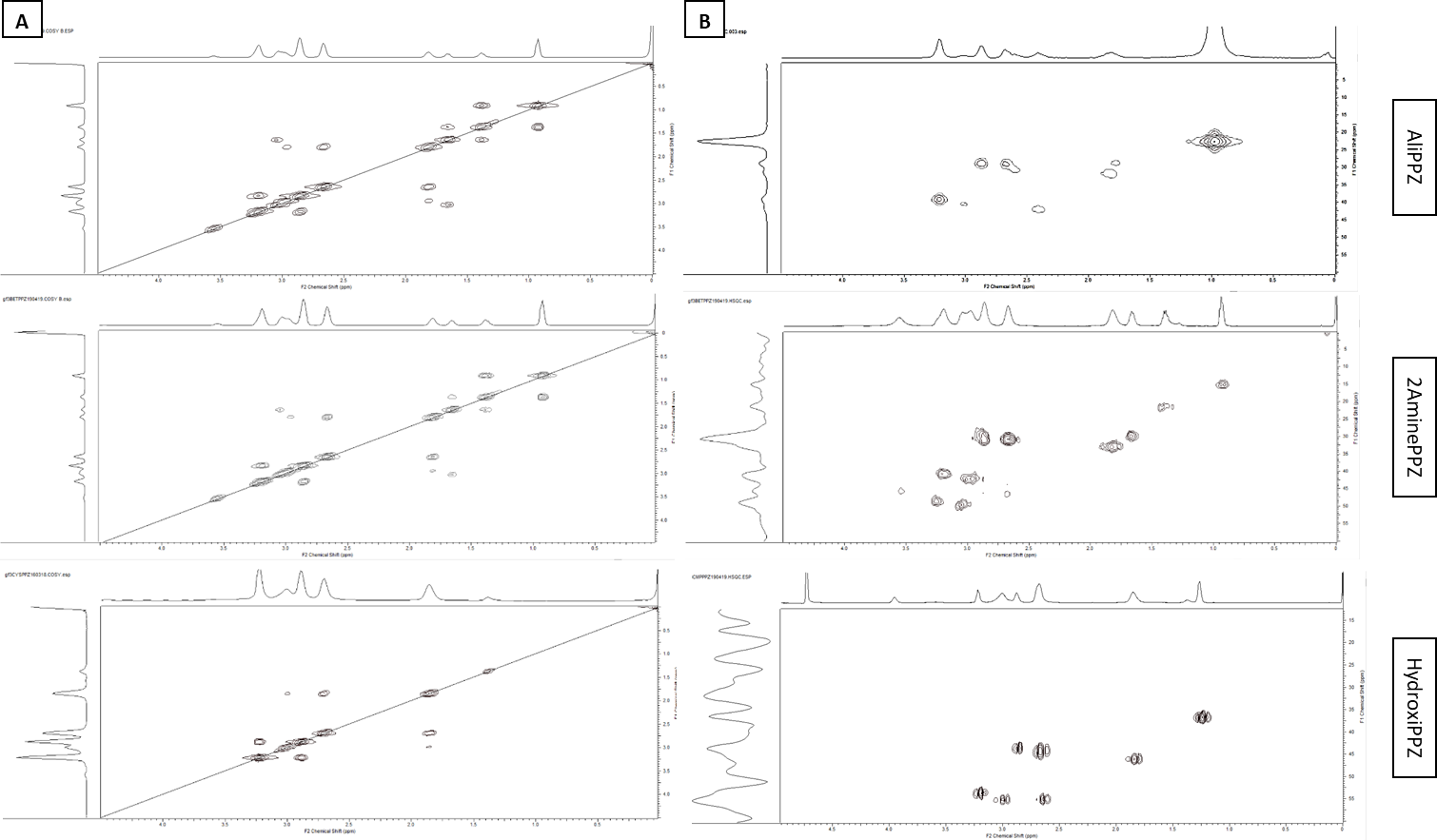

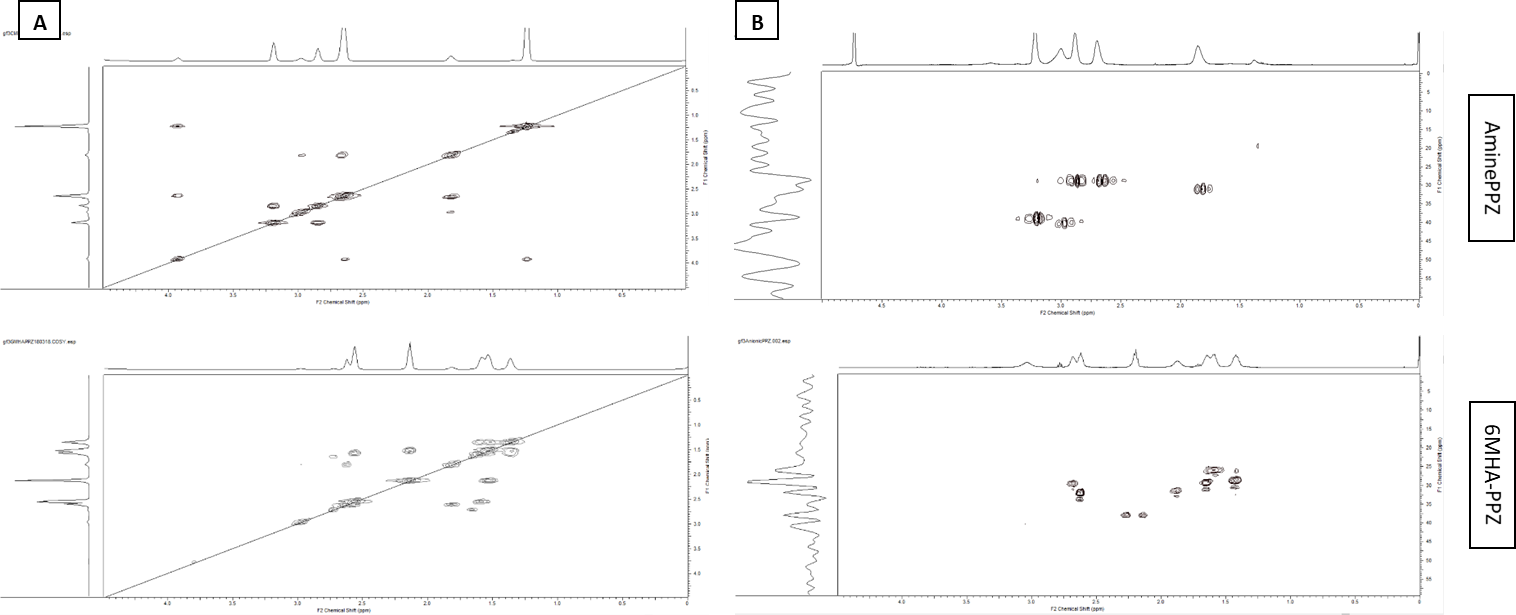


S5. Determination of polymer molecular weight. Fractograms of Asymetric Flow Field-Flow Fraction (AF4) (n=2).


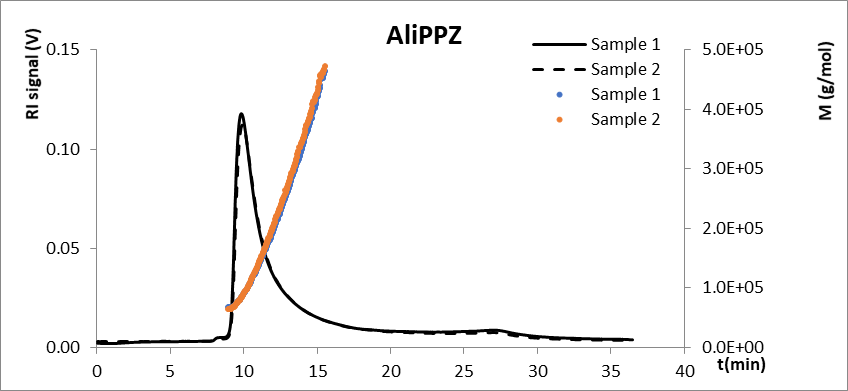


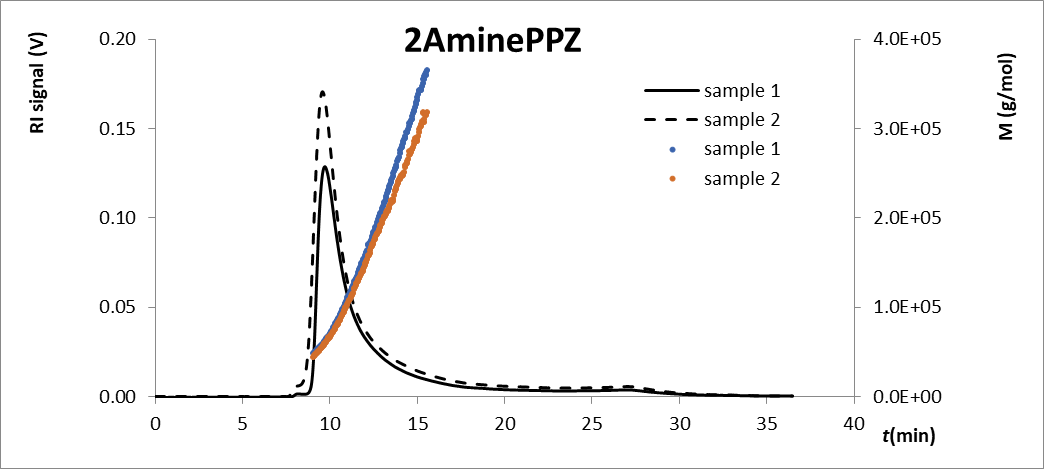

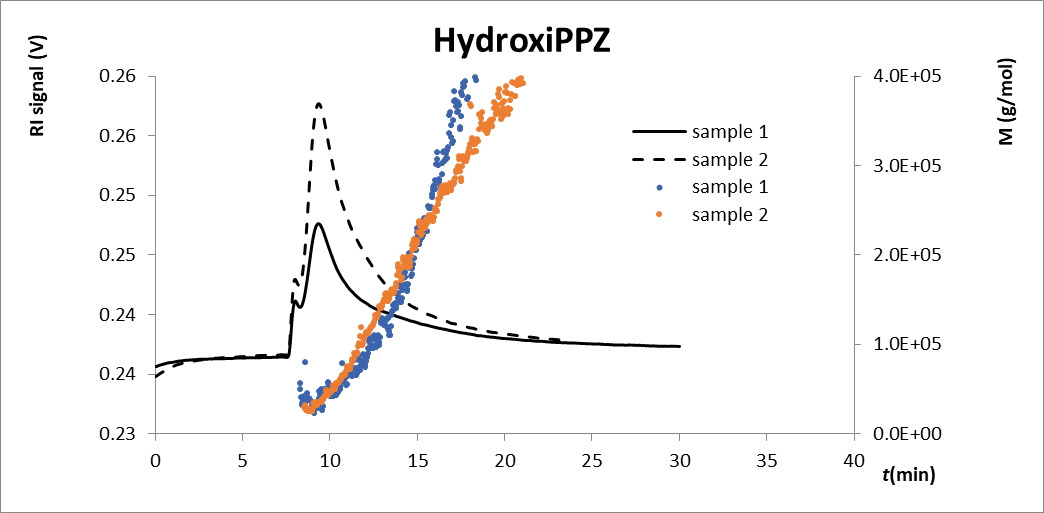


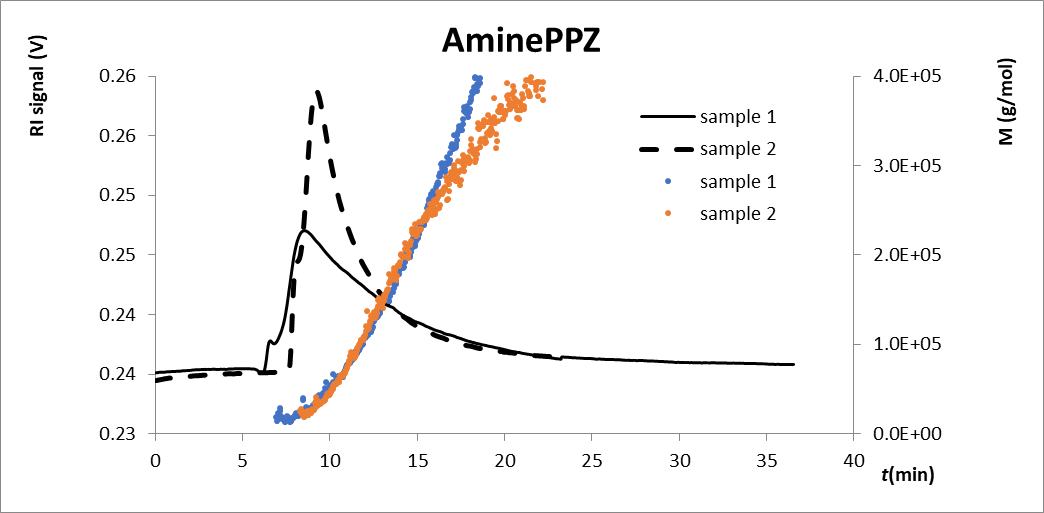

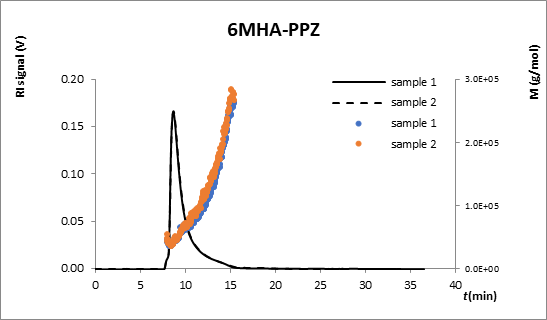


**S6. Cationic nanoparticles characterization by hydrodynamic size and zeta potential.**

| Cationic PPZ | Nanoparticles (N:P) | Size (nm) | PDI | Zeta Potential (mV) |
| --- | --- | --- | --- | --- |
| **AliPPZ** | 4:1 | 131±2 | 0.2 | +46±3 |
|  | 8:1 | 141±4 | 0.2 | +54±1 |
|  | 16:1 | 149±6 | 0.3 | +54±2 |
| **2AminePPZ** | 4:1 | 96±7 | 0.2 | +48±3 |
|  | 8:1 | 110±5 | 0.1 | +53±4 |
|  | 16:1 | 122±5 | 0.2 | +56±2 |
| **HydroxiPPZ** | 4:1 | 101±3 | 0.1 | +31±2 |
|  | 8:1 | 116±2 | 0.1 | +34±4 |
|  | 16:1 | 117±3 | 0.1 | +41±3 |
| **AminePPZ** | 4:1 | 110±7 | 0.2 | +46±2 |
|  | 8:1 | 129±7 | 0.1 | +51±1 |
|  | 16:1 | 128±5 | 0.2 | +54±1 |

**S7. Relationship between size and nanoparticle concentration.**


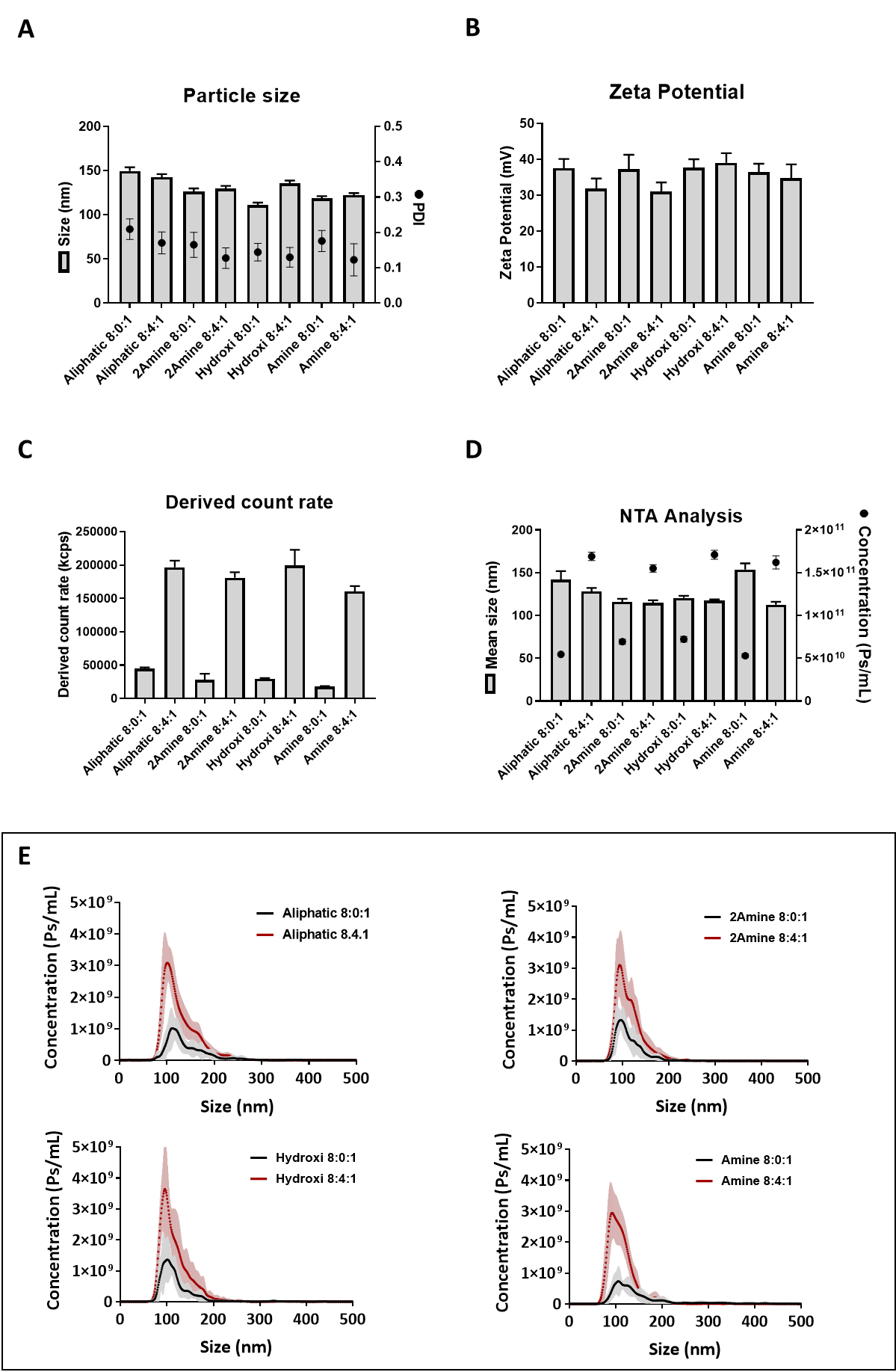


**S8. Nanoparticle morphological characterization by electronic microscopy FESEM.** Transmission electron microscopy (grey images) and scanning electron microscopy (black images).


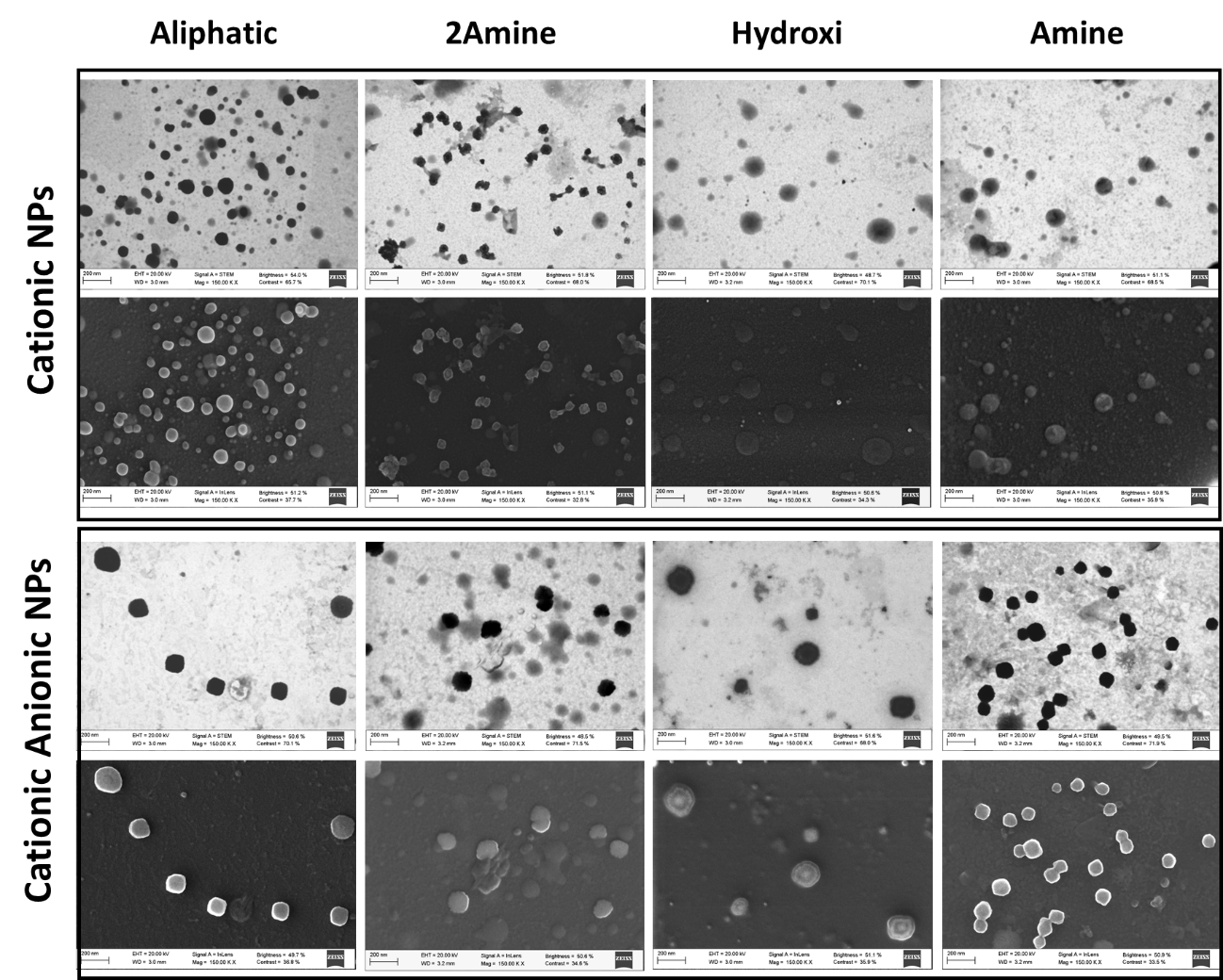


**S9. Representation of cell viability at different concentrations of nanoparticles.** A. Viability determination based on cell metabolic activity of human glioblastoma cell line (U87MG) treated with different concentrations of nanoparticles (ratio 8:0:1 and 8:4:1) expressed as μg pDNA / cm^2^ at 48 h post-treatment. B. Correlation between LC50 expressed as μg pDNA / cm^2^ and μg cationic polymer / mL.

**A**


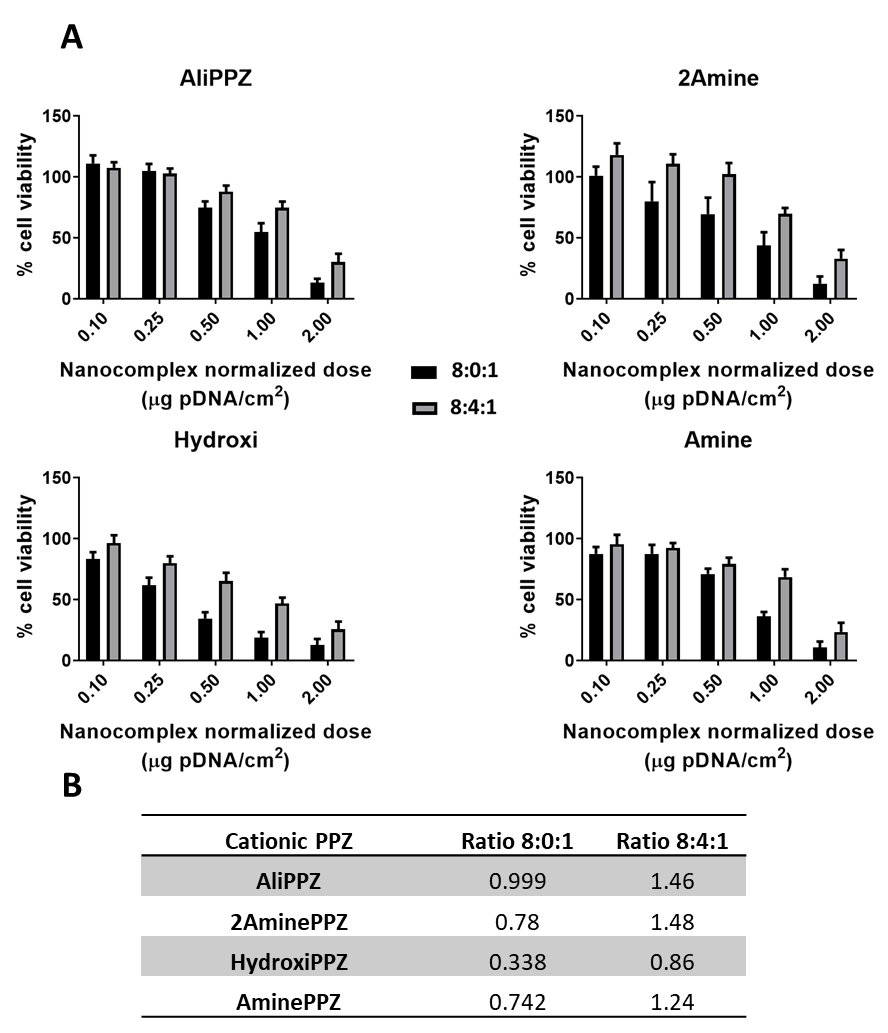


| Cationic Polymer  **B** | Ratio | LC50 expressed as μg pDNA / cm^2^ | LC50 expressed as μg cationic polymer / mL |
| --- | --- | --- | --- |
| AliPPZ | 8:0:1 | 1.00 | 12.54 |
|  | 8:4:1 | 1.46 | 18.31 |
| 2AminePPZ | 8:0:1 | 0.78 | 5.59 |
|  | 8:4:1 | 1.48 | 10.61 |
| HydroxiPPZ | 8:0:1 | 0.34 | 4.18 |
|  | 8:4:1 | 0.86 | 10.57 |
| AminePPZ | 8:0:1 | 0.74 | 4.55 |
|  | 8:4:1 | 1.24 | 7.62 |

**S10. 3D toxicity assay, evolution of the neurosphere area after the treatment with the different prototypes.**


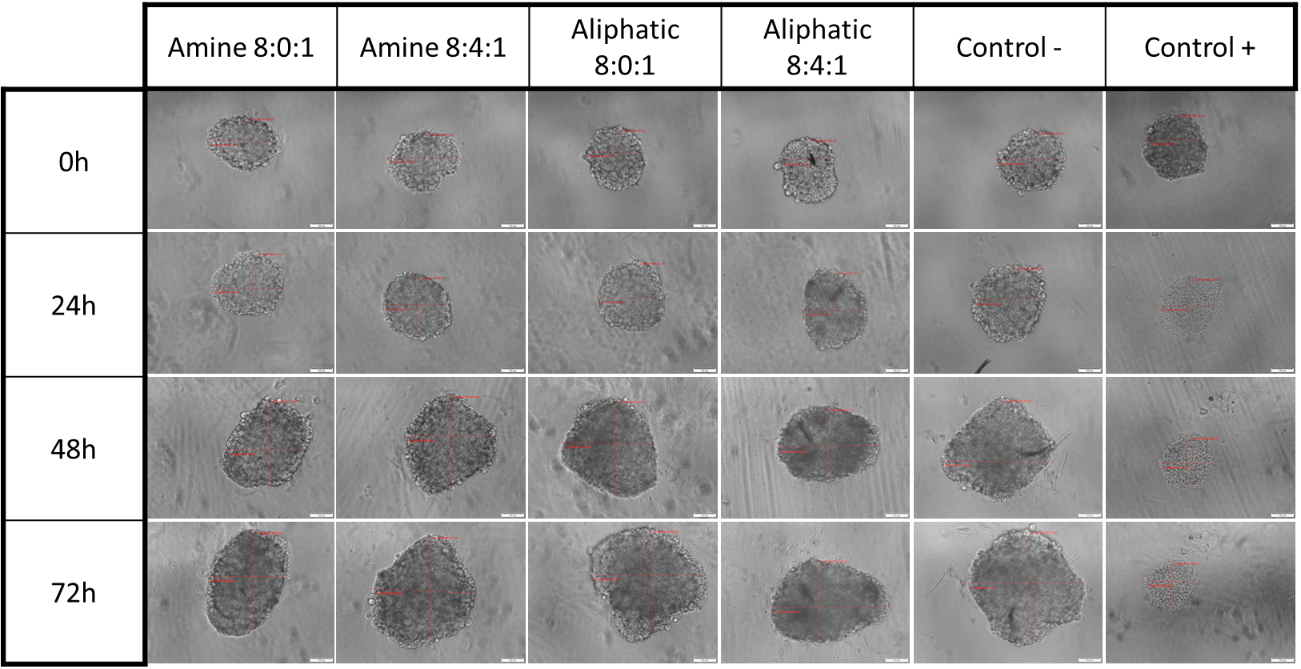


**S11. Survival of the introduced Danio rerio as observed under presence of the nanocomplexes at different times**. The concentrations are expressed in μg of cationic polyphosphazene/mL. Control of survival: osmosis water.


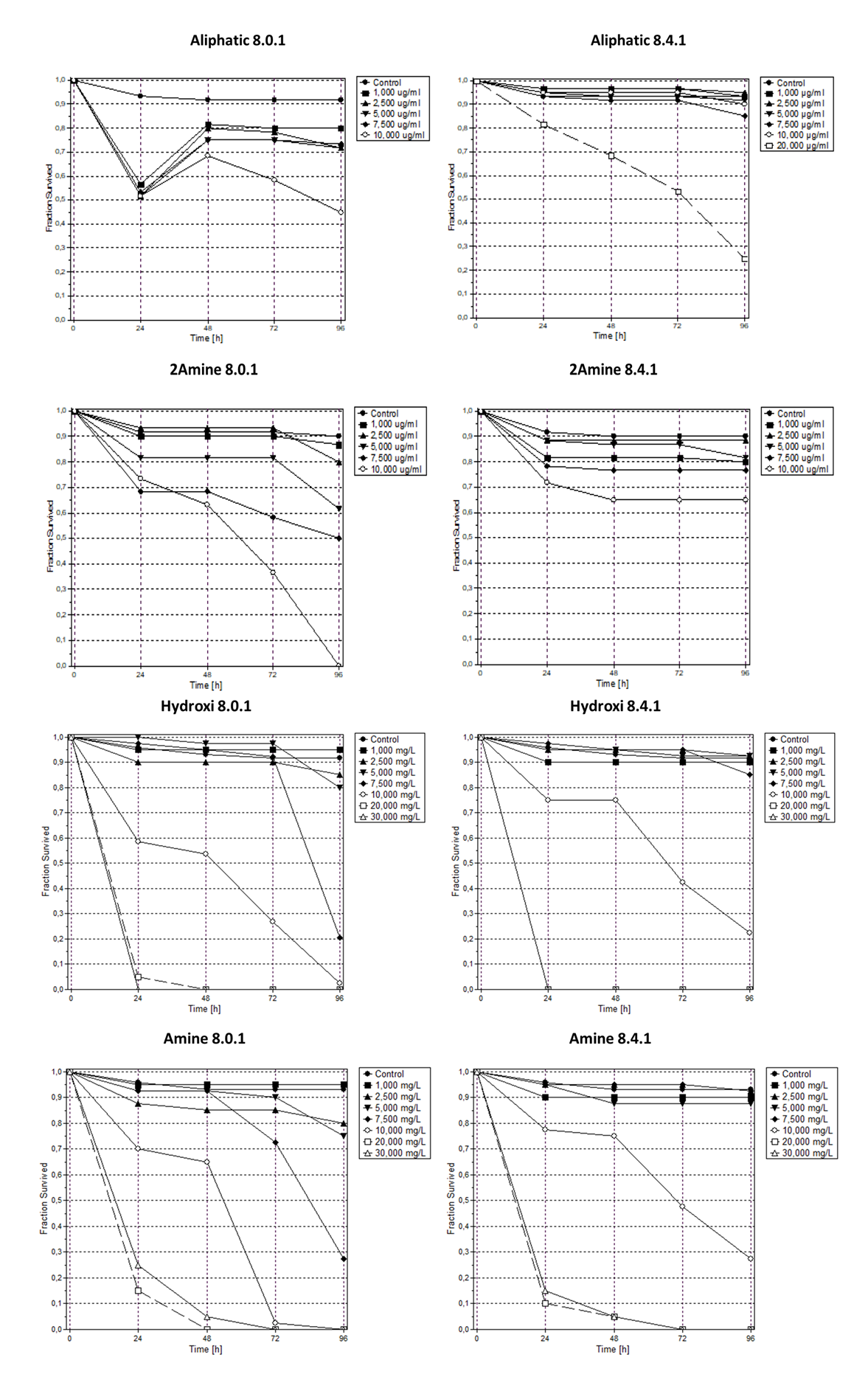


**S12. Qualitive transfection of the Cationic/Anionic NPs by green fluorescence microscopy after 48 h pos-treatment.**


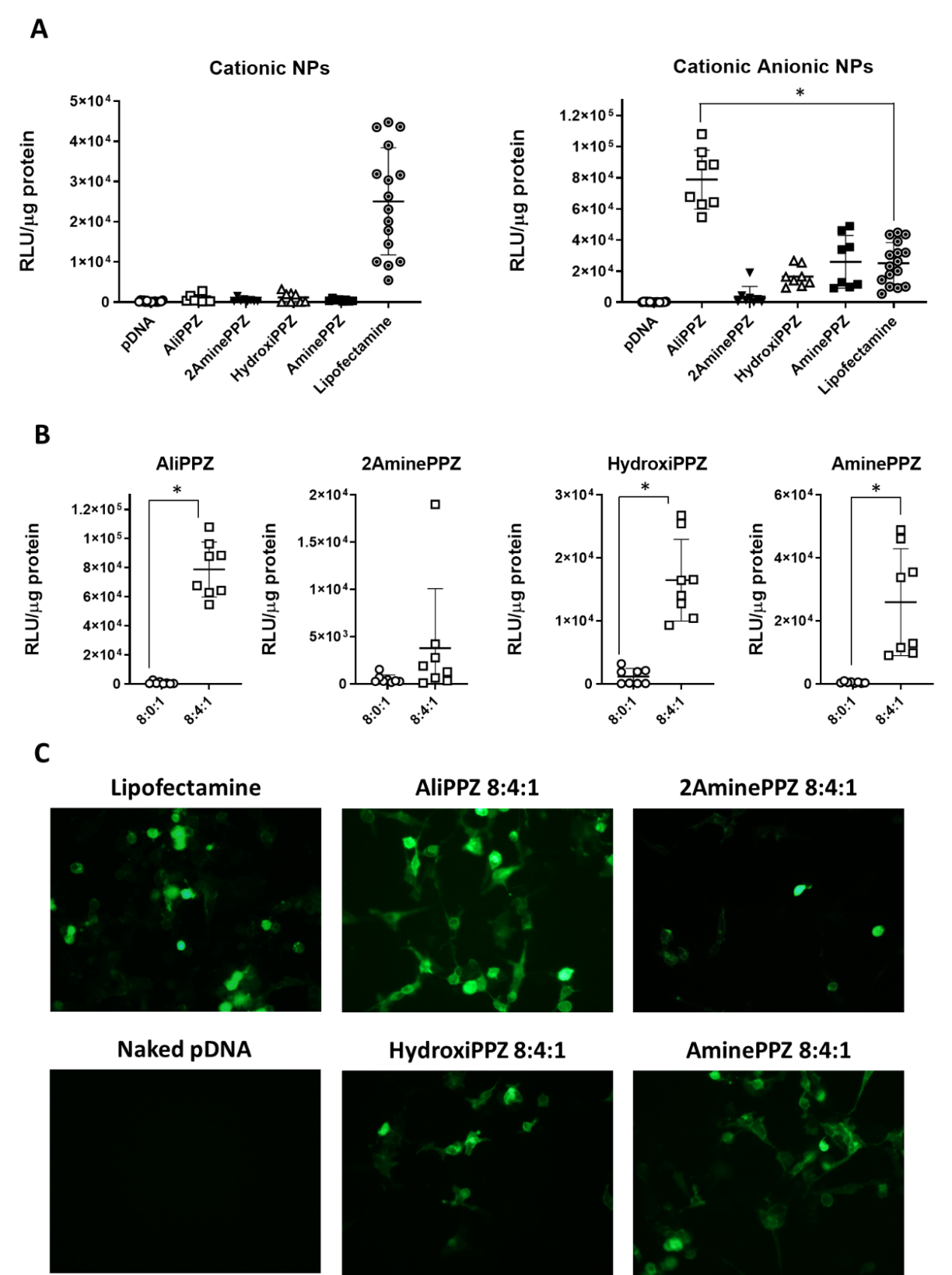


Figure S13. Characterization of therapeutic nanoparticles (pBMP4-NPs). A. Nanoparticle size and polydispersity of nanoparticles prepared with therapeutic plasmid. B. Gel retardation assay to test the association of the nucleic acid and the dissociation in presence of a competitor. NPs: nanoparticles, Hep: heparin.


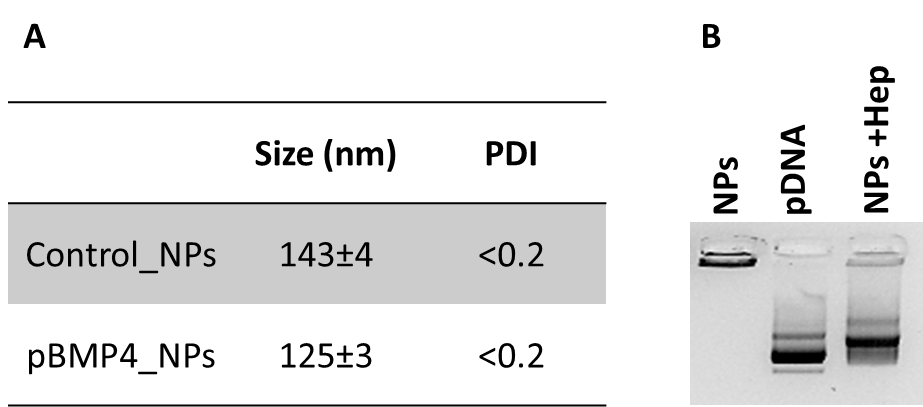


**Figure S14. Efficacy in vivo of the formulation alone and in combination with a chemotherapeutic agent**. a. Evolution in the weight of the mice during the experiment. b. Evolution of tumor volume corrected for initial volume. The red arrows illustrate the four administrations on consecutive days. Tz: Temozolomide, NPs: Nanoparticles; BMP4: Bone morphogenic protein 4.


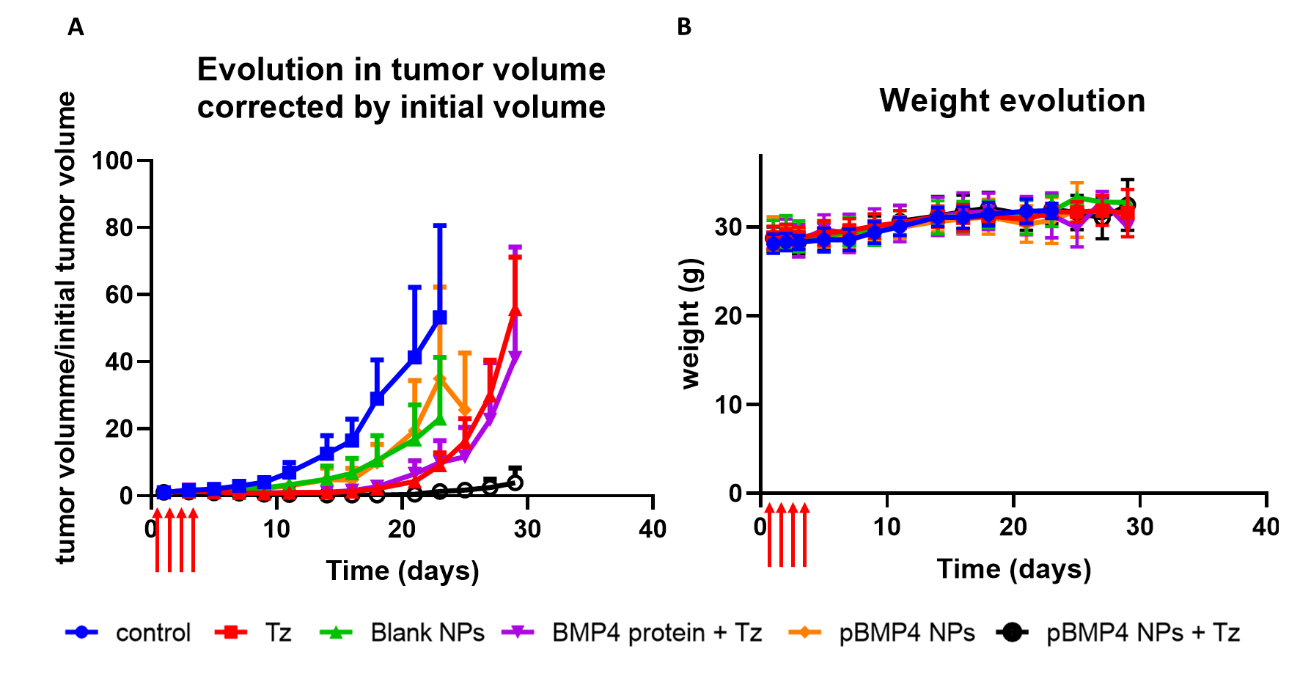


**Figure S15. Expression of RNA corresponding to cancer stem cell markers and efflux pumps determined by Real time-PCR.**


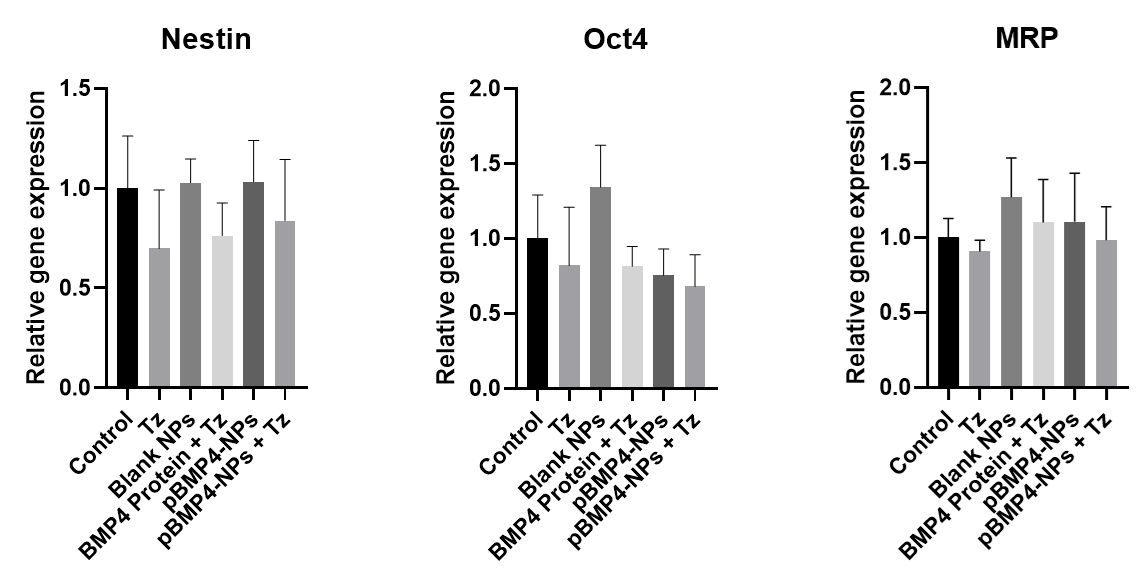

Supplement: Multimedia component 1 [file mmc1.docx]
